# Snowflake Model of Water: A Fast Approach for Calculation of Structural Properties of Liquid Water

**DOI:** 10.1021/acs.jctc.5c00158

**Published:** 2025-05-12

**Authors:** Peter Ogrin, Tomaz Urbic

**Affiliations:** Faculty of Chemistry and Chemical Technology, University of Ljubljana, Vecna Pot 113, SI-1000 Ljubljana, Slovenia

## Abstract

We develop a statistical–mechanical model to calculate the structural properties of liquid water. The model is based on the generation of snowflake-like structures that serve as an approximation for the structure of liquid water. It is a two-dimensional model in which each water molecule has three interaction sites that can form three types of interactions, namely, hydrogen bonding, van der Waals contact, and no interaction. The structural model is based on the analytical water model—UD model used for the prediction of thermodynamic and dynamic properties; however, the UD model is not able to predict structural properties. Here, the UD model was adapted to match the properties of the rose water model used in the simulations. The thermodynamic and dynamic properties calculated with the adapted UD model and the simulations of the rose model are in good agreement. The new snowflake model was used to calculate the structural properties of water. With this model, we calculated the radial and angular distribution functions of the water molecules and compared them with the functions from the simulations of the rose water model. The snowflake model was very successful in reproducing the functions calculated from the simulations. In addition, the spatial distribution functions were calculated with the snowflake model. Altogether, the UD model and the snowflake model allow us to calculate the thermodynamic, dynamic, and structural properties of liquid water with comparable accuracy to the simulations, but only for a fraction of the calculation time.

## Graphical abstract

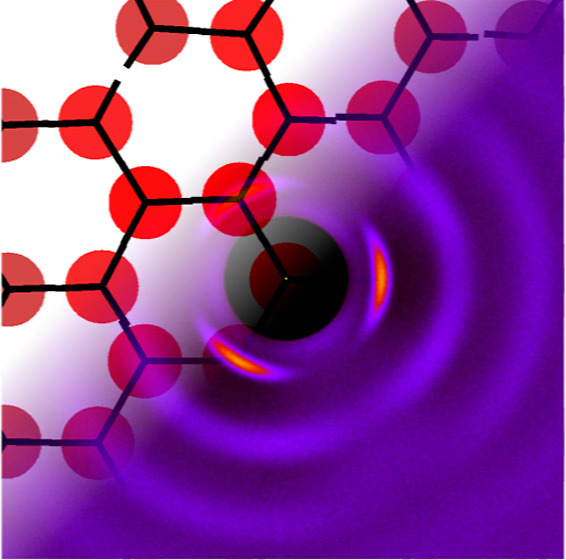

## 1 Introduction

The properties of water and aqueous solutions have been widely and extensively researched, as scientists and researchers have been aware of the importance of water in many living and nonliving systems since the early days of science. The importance of water arises from its abundance in combination with its anomalous properties,^1^ which affect practically all systems in which water is involved. The anomalies exhibited by water are thermodynamic (such as the density maximum at 4 °C, the negative thermal expansion coefficient, and the unusually high heat capacity in the liquid state), dynamic (such as the anomalies in the diffusion coefficient^2–7^ ), and also structural anomalies (such as a rich and complex phase diagram with 20 solid ice phases^8–10^ and anomalous regions of local structural ordering^11^). Although there are many different anomalous properties of water, the origin of most of these anomalies lies in the ability of water molecules to form hydrogen bonds. The tetrahedral local structure into which the water molecules are organized is thought to be associated with many of the anomalies of water.^12,13^ This link between the tetrahedral structure and the anomalous behavior is supported by the fact that similar anomalous properties found in water are also found in other liquids that exhibit a tetrahedral structural order.^14,15^ Such liquids are, for example, liquid *GeO*_2_,^16^ liquid *BeF*_2_,^17,18^ liquid silicon,^19^ and liquid silica.^20,21^ It therefore appears that the local structure of water plays an important role in practically all of the properties of liquid water. When water is confined on the nanoscale, its properties differ from those of bulk water. For example, when water is confined in a monolayer, its dielectric constant decreases;^22^ the phase behavior of water monolayers is quite rich and differs from that of water in the bulk,^23–29^ the confinement also changes the freezing point,^30,31^ increases diffusion.^32^ Water confined in a layer still shows similar anomalous thermodynamic properties as bulk water, but they are shifted toward lower temperatures, while the structural and dynamic properties are similar to bulk water at higher temperatures.^33^ Ab initio calculations have shown that the structures that form in water belong to two families characterized by a square or honeycomb hydrogen bonding network.^34^ Water monolayer also undergoes a liquid–liquid phase transition at low temperatures.^35^ Overall, it appears that confinement of water in a monolayer changes its properties quantitatively, for example, phase transitions, anomalies, etc., shifting to other conditions. Qualitatively, however, water still shows the same fundamental properties that make water such an interesting anomalous liquid, as these properties are consequences of water’s ability to form hydrogen bonds. In this work, we have used 2D models, which in principle can also be used for modeling monolayers. However, the models used in this work, and the Mercedes–Benz (MB) model that inspired them all, have never been used to model monolayers and have never been tested to see whether they actually capture the properties of monolayers. One of the key properties of monolayers is their confinement, which restricts the movement and degrees of freedom of the molecules. Since the 2D water model is not confined in any way in 2D space, such a model cannot automatically be interpreted as a water monolayer. Instead, such models have always been used as a 2D simplification of water, which still has properties similar to those of physical (3D) water.

Due to the importance of water in different systems, many water models have been developed to predict or explain the different properties of water in different environments. The first realistic water model was the Bernal–Fowler model,^36^ in which water molecules were modeled with point charges and a repulsion–dispersion term. Later, a similar model was used in the first Monte Carlo (MC) simulations of water.^37^ With the development of computer technology, new models for water were developed. Very popular groups of models that were developed quite early in the history of water models but are still frequently used today are the models of the groups TIP^38–43^ and SPC.^44,45^ All of these models have the same basic structure in the sense that they contain combinations of Lennard–Jones (LJ) potential and point charges. These models are still used today and are very popular for the simulation of more complex systems (such as solutions of biomolecules) in which water acts like a solvent. Over time, many different water models have been developed, differing in which properties and systems they specialize in, as well as in their complexity. Nowadays, the computing power of computers is so great that, in combination with machine learning methods, even ab initio quantum mechanical methods can be used to calculate the properties of water.^46^ With such computationally intensive models, many different properties and effects can be included in the calculations that would otherwise be neglected (e.g., nuclear quantum effects).

On the other side of the complexity spectrum, there are simple models that consider only the most important properties of water and may even lack atomistic details. The advantage of such simple models is that they can be used to investigate the fundamental physical causes and mechanisms behind various phenomena. In this way, the relationships between the properties under investigation become clearer and free from the interfering effects of other interrelated factors. Due to their simplicity, simple models can also be used to develop and improve various more or less complex methods, ranging from complex machine learning algorithms to simple or complex statistical–mechanical theories. If the simple model is 2D, it also enables easier visualization of the structure so that we can see various changes and effects more clearly. Examples of such simple 2D models are the MB model^47,48^ and the rose water model.^49^ These two models are quite similar, as the rose model was developed as a computationally more efficient mimic of the MB water model. Another model based on the same idea as the MB and rose models is the coarse-grained water model mW.^50^ Similar to the MB model, this model does not consider long-range electrostatic interactions but instead explicitly uses hydrogen bonding by favoring the tetrahedral arrangement of the molecules. There is also a newer, coarse-grained model that uses the idea of explicit tetrahedral hydrogen bonding.^51^ In this work, we have used the rose water model, which, as mentioned earlier, is a simple 2D water model that represents the molecules as discs interacting through the LJ potential and additional explicit hydrogen bonding potential. This model successfully reproduces crucial anomalous properties of water and has already been used in simulations;^52–55^ moreover, due to its simplicity, the rose model has also been used in statistical–mechanical theories such as integral equation theory and thermodynamic perturbation theory.^52,56–59^ The statistical–mechanical theories enable the calculation of properties with less computational cost than simulation. This is a great advantage that allows us, for example, to investigate a wider range of conditions under which the properties of the system may differ, e.g., if we want to determine a phase diagram, the simulations are very time-consuming.^60^ However, the disadvantage of the theories is that they use some approximations that may affect the accuracy of the results. Another disadvantage is that different theories are used to calculate different properties. For example, thermodynamic perturbation theory can only provide thermodynamic results and, thus, cannot give us information about the structure of the system. Another example from our previous studies is that the orientation-averaged integral equation theory, which is the most commonly used, gives us orientation-averaged results, although the actual structure depends on the orientation. We can use the orientation-dependent version of integral equation theory to get orientation-dependent results, but the orientation-dependent version is computationally much more expensive than the orientation-averaged version; the computational time required is almost on the order of simulations. So, even if we have relatively simple models that can be used in various statistical–mechanical theories, they are still quite time-consuming. The next step on the way to faster calculations is therefore the creation of analytical models that can be used for the direct calculation of various properties.

There have been several attempts to develop an analytical water model.^61–64^ Based on the MB water model, an analytical water model has been developed,^65,66^ which can be categorized as a variable-structure cell theory. In these models, each molecule is confined by neighboring molecules in a cell. The cell has three possible states: cage-like, dense, and extended. These states represent the mutual interaction of the molecule in the cell with two neighboring molecules. Based on this treatment, a simpler analytical model was later developed by Urbic and Dill (UD model).^67^ This UD model uses only two-body interactions, which makes it simpler, and ice has a higher multibody cooperativity. Overall, the derivation of the partition function for this model is more intuitive and simpler. This analytical UD model can be used to calculate various thermodynamic properties of water. Recently, the UD model has been extended and further developed so that it also enables the calculation of the dynamic properties of water, such as the diffusion coefficient, viscosity, and thermal conductivity.^68^ The model for the dynamic properties is based on the idea of random walk. The reason why a 2D model is used as the basis for developing an analytical model is that the 2D model is easier to visualize, and it is therefore also easier to develop the analytical model on this basis. Later, when the concept of the model was perfected in an optimal form, we planned to extend the model into three dimensions.

In this work, we have further developed the UD analytical model so that it now enables the calculation of the structural properties of water. The new model—snowflake model is based on snowflake-like, which are generated as approximations of the differently ordered water molecules in the system. The analytical water model is now complete in the sense that it enables the calculation of all types of water properties: thermodynamic, dynamic, and structural. The details of the model and the methods used can be found in Section 2. To test the new model, we parametrized the UD model to resemble the properties of the rose water model. We then calculated the full profile of the model properties and compared it with simulations of the rose water model. Therefore, in the Results and Discussion section (Section 3), you will first find a relatively brief comparison of the thermodynamic and dynamic properties of the analytical model and rose model used in the simulations. This is followed by a somewhat more detailed section in which the structural properties calculated with the analytical model and simulations are presented and compared. At the end, the work is summarized in Section 4.

## 2 The Model

The snowflake model used here is based on the UD model that was originally developed for the calculation of the thermodynamic properties of water^67^ and later improved so that it can also be used for the calculation of the dynamic properties of water.^68^ Here, we have used the modification of the aforementioned UD model to calculate the thermodynamic and dynamic properties. However, to calculate the structural properties of water, we developed a new model based on the parameters and principles of the UD model.

### 2.1 Statistical Mechanics of the Model

Let us first briefly describe the UD model used to calculate the thermodynamic and dynamic properties of water. More detailed summary of the model can be found in Supporting Information, while all the details about this model can be found in ref 67. In this model, it is assumed that there are *N* molecules in the system. The theory focuses on the single water molecule forming a hexagon with other molecules and on the relationship of this molecule to its neighboring molecules. The molecule has three interacting arms/thirds of the molecule that can interact with other molecules and form hydrogen bonds. Since the arms are equivalent, the 3-fold symmetry is taken into account. There are three possible interaction states (Figure 1) of the arm (third of the molecule), namely, HB, vdW/LJ contact, and no interaction at all (0). For each of these interactions, the isothermal–isobaric statistical weight of the state can be calculated.

**Figure 1:**
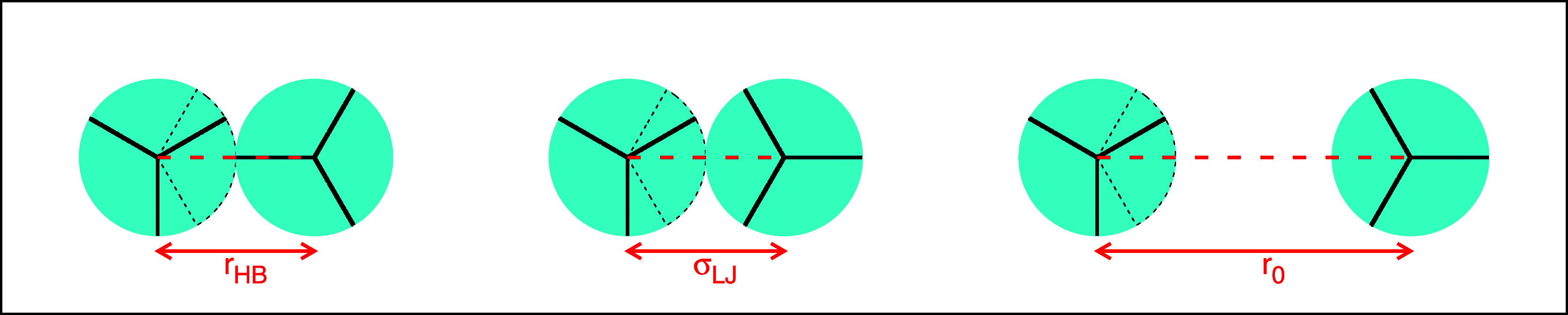
Schematic representation of three possible interaction states: HB, LJ, and noninteracting state.

#### 2.1.1 The Hydrogen-Bonded State

In the hydrogen-bonded state, the central molecule is oriented so that one of its three hydrogen-bonding arms points to the center of the neighboring water molecule, while the angle between the arm and the line connecting the centers of the interacting molecules is within π/3. The energy of such an interaction of the central molecule with its neighbor is as follows

1 $$u_{HB}\left(\theta \right)=-\varepsilon_{HB}-\varepsilon_{LJ}+k_{s}\theta^{2},,-\pi /3<\theta <\pi /3$$

where ε_HB_ is the HB energy constant representing the maximum strength of the HB, ε_LJ_ is the LJ contact energy constant representing the vdW part of the energetic interaction, and, finally, *k*_s_ is the angular spring constant added to model the angular dependence of the HB. This type of HB represents a singular HB (later we also introduce cooperative HB).

#### 2.1.2 The vdW/LJ Contact State

The second state in which a pair of interacting molecules can be in is a state in which the molecules are in direct van der Waals or LJ contact but also do not form hydrogen bonds with each other. The energy of such an interaction is

2 $$u_{LJ}=-\varepsilon_{LJ}$$

#### 2.1.3 The Noninteracting State

The third state is a state that represents molecules that do not interact; therefore, its energy is

3 $$u_{0}=0$$

#### 2.1.4 The Partition Function

Next, isothermal–isobaric partition functions are calculated for each interaction state and then combined into the partition function of a whole hexagon consisting of six water molecules:

4 $$Q_{1}={\left(\Delta_{HB}+\Delta_{LJ}+\Delta_{0}\right)}^{6}$$

This equation assumes that the molecules in the hexagon are independent of each other. In ice, however, there is a higher degree of hydrogen bonding cooperativity between the molecules and not just pairwise hydrogen bonding. Therefore, the solid state - ice, in which the entire hexagon forms hydrogen bonds, becomes more favorable by changing the partition function of the hexagon:

5 $$Q_{1}={\left(\Delta_{HB}+\Delta_{LJ}+\Delta_{0}\right)}^{6}-\Delta_{HB}^{6}+\delta \Delta_{s}^{6}$$

where δ = exp­(−βε_c_) is the Boltzmann factor for the cooperative energy ε_c_. The difference between Δ_HB_ and Δ_s_ is that in Δ_s_, *v*_s_ is used instead of *v*_HB_, since this state represents a state in which all molecules consist of HB and thus of a perfect hexagonal lattice. Consequently, the partition function for the entire system of *N* molecules is *Q* = *Q*_1_^*N*/6^. Various thermodynamic properties can be calculated from the partition function. The effects of different conditions on the system are not assumed in advance but result from the partition function. The interactions can be in one of 4 states, which are used in the partition function of the hexagon. Important quantities that are used later in the structural model are the populations of the states *i* = 1 (HB), 2 (LJ), 3 (0), and 4 (s), which are calculated as follows:

6 $$f_{i}=\frac{\partial \log Q_{1}}{\partial \log{\;\Delta }_{i}^{6}}$$

Other thermodynamic quantities are calculated using standard relations and are described in refs 65 and 67 .

### 2.2 Model for the Dynamic Properties

The model for dynamic properties is an extension of the UD model that was just described. We briefly summarize it here; more details can be found in Supporting Information and in an earlier paper.^68^ The main idea of the model is that the transport of molecules in the liquid and gas phases is similar to a random walk, i.e., their motion can be approximated by a random walk. Therefore, the diffusion of molecules in 2D is approximated by

7 $$D\propto \lambda^{2}\nu$$

where λ is the step size and ν is the step frequency. The total diffusion coefficient of water is calculated as a weighted average over different interaction states

8 $$D=T\sum f_{i}D_{i}$$

where *D*_*i*_ = λ_*i*_^2^ν_*i*_ represents the diffusion coefficients for different states, namely, HB, LJ, 0, and s. The step sizes for these states are approximated differently: the step sizes of the HB and s states are approximated by the length of the HB, λ_HB_ = λ_s_ = *r*_HB_, the step size of the LJ state is the LJ contact distance, λ_LJ_ = σ_LJ_, and for the noninteracting state, the average distance between the molecules in this state is used, $\lambda_{0}=\sqrt{v_{0}}$ . The Boltzmann factor of the average energy of the states is used for the step frequency

9 $$\nu_{i}=C\exp\left(\beta \left\langle u_{i}\right\rangle \right)$$

where *C* is the constant responsible only for the units.

### 2.3 Snowflake Model—A Model for Structural Properties

Here, we developed a structural model of water that takes parameters and calculated quantities from the model described above and uses them to calculate the structural properties of water. The structural model is fractal-like (but mathematically not an actual fractal) and was inspired by the formation of snowflakes in nature. Similar to the UD model described above, the snowflake model focuses on the central molecule and the relationship between this molecule and its neighboring molecules. The main principle of the snowflake model is to create snowflake-like structures of water molecules that approximate the actual structural arrangement of water molecules. Here, we mimic the structural arrangement of water molecules, especially in the liquid state. This is done by a more or less disordered hexagonal hydrogen bonding network that resembles the structure of a honeycomb, which has also been found in water monolayers.^24,34^ In the paper, a pressure of 0.19 is used in reduced units, which corresponds to about 0.6 Pa in SI units, if we assume that the energy of HB is 120 kJ/mol and the length of HB is 0.25 nm. The crystal structure at this pressure is consistent with the phase diagram from first-principles calculations.^24^ However, it is important to note that the models we used are not parametrized to actual physical water, so they cannot be expected to quantitatively reproduce the properties of actual water. The pressure of 0.19 was chosen because it has been used in many similar studies using similar models. Since this model is built on the quantities from the UD water model, it must follow the same main assumption, namely, that the molecule has three interacting arms and that each arm can form three types of interaction: hydrogen bonding (HB state), LJ contact (LJ state), and no interaction (noninteracting state). The populations from eq 6 are taken here as probabilities that an arm forms each type of interaction with the neighboring molecule. The fourth type of interaction—the cooperative HB (solid (s) state)—is implicitly considered here, its population, *f*_s_, is added to the probability for an arm to form a HB, so that due to the cooperative energy, the overall probability of HB formation, *f*_HB_ + *f*_s_, is higher than it would be if the effect of HB cooperativity was not considered in eq 5 and eq 4 was used instead. In other words, the cooperative energy increases the population of the s-state (*f*_s_) because it makes it energetically more favorable. Consequently, it also increases the probability of HB formation because more molecules in the *f*_s_ state mean that there are more HBs in the system. Since this cooperative energy has already been taken into account in the calculations of *f*_s_, we do not need to consider it again here when determining the probability of adding a new molecule with each interaction type. Thus, when a new molecule is added to the snowflake, the probability of the new molecule to form the HB interaction is *f*_HB_ + *f*_s_, for the LJ interaction, it is *f*_LJ_, and for the noninteracting state, it is *f*_0_.

The algorithm starts with a central molecule at coordinates (0,0), this central molecule, *i* = 0, has three independent arms, and for each arm, *k*, *k* ∈ {0, 1, 2}, an interaction type, *j*, is randomly selected according to the population probability *f*_*j*_, *j* ∈ {HB, LJ, 0}, where in HB interaction both HB and *s* states are included. The next shell of molecules is then added to the snowflake using a simple geometry:

10 xi,k=xi−1+cos(θi−1+kq+Zθ,j)(rj+Zr,j)yi,k=yi−1+sin(θi−1+kq+Zθ,j)(rj+Zr,j)θi,k=θi−1+kq+Zθ,j+π

where *x*_*i*,*k*_, *y*_*i*,*k*_, and θ_*i*,*k*_ are coordinates of the new molecule, *q* is the angle between the arms (120°), and *k* is the arm index. The central molecule can form 3 arms, so that for the first shell, the *k* goes from 0 to 2, while for the other shells, the *k* goes from 1 to 2, since the arm 0 already connects the molecule in shell *i* – 1 with shell *i* – 2. The *r*_*j*_ are the bond/interaction lengths for each type of interaction: *r*_HB_ = *r*_HB_, *r*_LJ_ = σ_LJ_ × 2^(1/6)^ and $r_{0}=\sqrt{T/p}+r_{LJ}$ . *Z*_*r*,*j*_ and *Z*_θ,*j*_ are parameters that take into account the random motion of the molecules:

11 $$\begin{array}{l} Z_{r,HB}∼N\left(0,{\left(\sqrt{\frac{k_{B}T}{m_{1}k_{HB}}}A\right)}^{2}\right)\\ Z_{\theta ,HB}∼N\left(0,{\left(\sqrt{\frac{k_{B}T}{m_{3}k_{HB}}}A\right)}^{2}\right) \end{array}$$

12 Zr,LJ∼N(0,(kBTm1kLJA)2)Zθ,LJ∼U(−π3,π3)

13 Zr,0∼N(0,(kBTm1k0A)2)Zθ,0∼U(−π3,π3)

where N and U are normal and uniform distributions, *T* is the temperature, *A* is the empirical coefficient for the scaling, *k*_*j*_ is the harmonic spring coefficient since each type of interaction is modeled with a harmonic potential (for HB, it is the same as in the thermodynamic part of the model), and *m*_1_ and *m*_3_ are the mass and moment of inertia of the water molecule, which in our case are 1 and 3 × 0.1 × 0.35^2^. The moment of inertia is calculated as the inertia of three arms, where the inertia of each arm is the square of the arm length multiplied by the arm weight. The variances of the normal distributions used to generate the random motion contributions were derived semiempirically. The potentials of each interaction are approximated by the harmonic potential *U* = *k*_*j*_ *x* ^2^/2 – ε_*j*_. By integrating the Boltzmann factor over all positions, the variance of the position $\sigma^{2}=\left\langle x^{2}\right\rangle =\frac{k_{B}T}{k_{j}}$ is obtained. The mass, the moment of inertia, and the empirical scaling coefficient *A* were then empirically added to the variance. The coefficients of the harmonic potential were determined in the following way: for HB, the coefficient *k*_s_ was taken from the thermodynamic part of the model, for LJ, the coefficient was obtained by fitting the harmonic potential to the LJ potential of the rose model, and for the noninteracting state, the coefficient was determined empirically so that the radial distribution functions calculated with the theory have the correct shape, which means that the RDF does not decrease with distance at high temperatures. The effects of the wrong coefficient *k*_0_ are shown in Figure S2. The constant *A* was adjusted in such a way that the height of peaks in the RDF calculated with theory was the same as the height of peaks from the simulations. The schematic representation of how new molecules are placed in the snowflake can be found in Figure 2.

**Figure 2:**
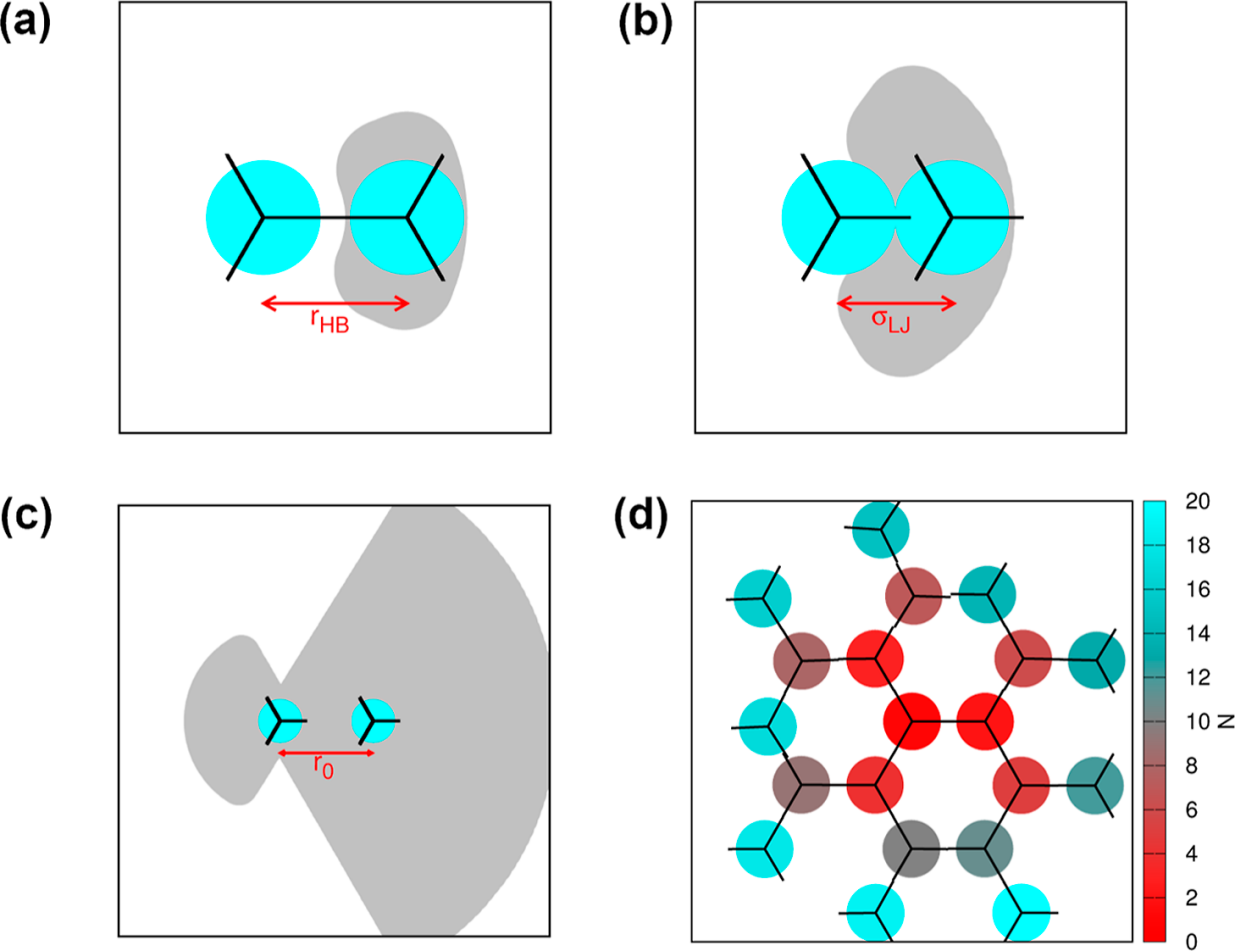
Schematic representation of the position of the new molecule added to the structure based on the three possible interaction states: (a) HB, (b) LJ, and (c) noninteracting state. The left molecule in pair is a source molecule, while the right molecule is the newly added one. Molecules are drawn with cyan color, the black lines represent HB arms, and gray area is a standard deviation of position of the new molecule. (d) The schematic representation of the growth of a snowflake-like structure in the ideal case, when all molecules form HB. A more red color represents earlier particles, while a more cyan color represents particles that were added to the structure later.

After the first shell of molecules has been created around the central molecules, the algorithm creates the second shell in the same way as the first shell. But now each molecule in the first shell can only form two new interactions, as one interaction is already used to interact with the central molecule. So only two new molecules are created from each molecule in the first shell to create the new shell of molecules, eq 10 is applied again. In this way, several shells of water molecules are created (Figure 2d). In our calculations, the maximum number of shells was set to 9. When new molecules are generated, the molecules are placed in such a way that they do not overlap. This means that when a new molecule is placed in the system, the algorithm checks the distances of the new molecule from all the molecules already in the system. If the distance to one of these molecules is less than σ_LJ_ – 0.05, the new molecule is not placed at this position. The algorithm then tries to place the same molecule (which is bound to the same source molecule by the same type of interaction) again. Due to the parameters of the random movement in eq 10, the position of each attempt is now slightly different from the previous placement attempt. The algorithm attempts to include the new molecule into the system until the inclusion is successful or the maximum number of attempts is reached. We have used 10 as the maximum number of attempts; this number is quite small, but it speeds up the algorithm without significantly worsening the results. If the maximum number of trials is reached without the new molecule being successfully added, then this branch of the snowflake is terminated. There is an exception to the condition that the molecules overlap when molecules of higher shell order (second and further shells) are placed near the central molecule. If an attempt is made to place the new molecule at a position that is closer to the central molecule than *r*_LJ_, the following acceptance probability is calculated exp­(−*k*_LJ_(*r* – *r*_LJ_)^2^/*T*) and according to this probability, the new molecule will or will not be placed at the attempted position. This change in the overlap condition was made to make the RDF continuous at small distances, as the RDF would otherwise be discontinuous or truncated at small distances when using a strict cutoff distance.

The construction of the snowflake ends when the maximum number of shells is reached or when all branches of the snowflake are terminated; there is no more room for the new molecules of the individual branches. To obtain good statistics, many such snowflakes are generated. In our calculations, we have used 100,000 replicas of snowflakes. Examples of actual snowflake-like structures obtained during the calculations can be found in Figure 3. Radial distribution functions, angular distribution functions, and spatial distribution functions are then calculated from the positions of the molecules in the snowflakes in a similar way as in standard simulations. In some respects, the new structural model is similar to MC simulations but is many orders of magnitude faster than standard MC simulations while producing comparable results. There is *C*_3_ symmetry in the system. This could be used so that the structure is calculated for only a third of the circle instead of the entire circle. Alternatively, this can also be used when averaging so that we would average three-thirds of the circle. This could speed up the calculation.

**Figure 3:**
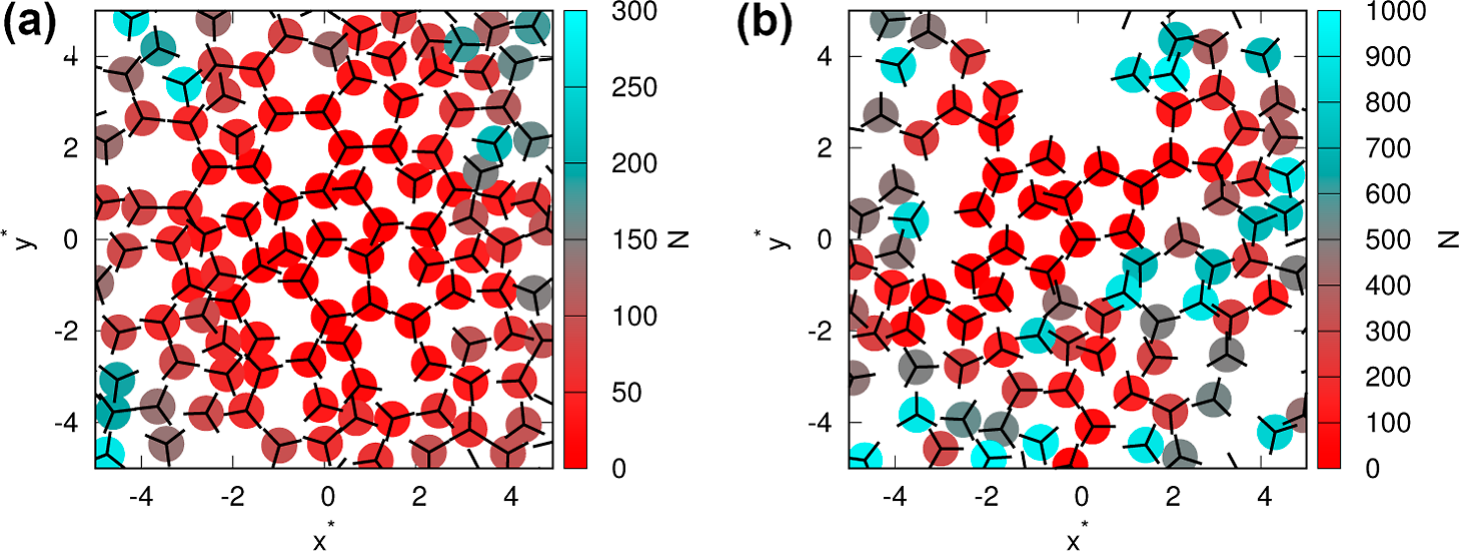
Examples of “snowflakes” at temperatures of 0.15 (a) and 0.25 (b), and the pressure is 0.19. The more red-colored particles represent particles that were introduced into the system earlier, while the more cyan-colored particles represent particles that were introduced into the system later.

In this work, we have used two parametrizations of the analytical model, both parametrizations serving to replicate the properties of the two parametrizations of the rose water model (MB and real parametrization). More details about the model and the two parametrizations can be found in the Supporting Information or in previous articles.^49,52^ In order not to confuse the reader with the original MB model, the parametrizations are also referred to as MB-rose and real-rose when we are talking about only the analytical model. For the thermodynamic part, the parameters of the models were determined by fitting the density as a function of temperature at a pressure of 0.19 to the density from the MC simulations. Care was also taken to ensure that the parameters are physically correct (e.g., that the density of the HB state (liquid) is not higher than the density of high-pressure ice (hexagonal lattice with filled interstitial sites)) and that the calculated thermodynamic functions are reasonable (we checked if other thermodynamic properties are qualitatively similar to the one calculated with simulations because there were combinations of parameters that produced excellent fit of density but other properties were qualitatively different from the ones from simulations). The quantities used for parametrization are part of the results and are shown in Figures 4 and S3. The HB and LJ energy and distance parameters are the same as those in the original rose model used in the simulations. Other parameters of the analytical model were *k*_s_ = 16, *a* = 0.06, ε_c_ = −0.05, and *x*_v_ = 1.0 for the MB parametrization and *k*_s_ = 6, *a* = 0.06, ε_c_ = −0.2, and *x*_v_ = 1.15 for the real parametrization. In the structural part of the model, the parameters were *k*_HB_ = 2*k*_s_ (the 2 comes from the fact that 1/2 is used in the harmonic potential in the structural part, but not in the thermodynamic part, but ultimately the potential is the same), *k*_0_ = 0.001 for both parametrizations, and *k*_LJ_ = 10.0, *A* = 0.650 for the MB parametrization and *k*_LJ_ = 12.0, *A* = 0.475 for the real parametrization. In the structural model, almost all parameters were taken from the thermodynamic model or determined by fitting to the original interaction potential (e.g., the constant of the harmonic potential for the LJ interaction). The only parameter that was adjusted according to the resulting properties was the constant *A* in eq 11, which determines the extent of the thermal random motion of the molecules. This constant was chosen so that the height of the main peaks in the RDF was the same when calculated with theory and simulation. The radial distribution functions for the fit were calculated at a pressure of 0.19 and a temperature of 0.2 for the MB parametrization. The parameters of the analytical model are shown in Table 1. Some of the parameters of the analytical model are the same as those of the rose water model used for the simulations. However, the analytical model is developed to describe properties of the rose water model.

**Figure 4:**
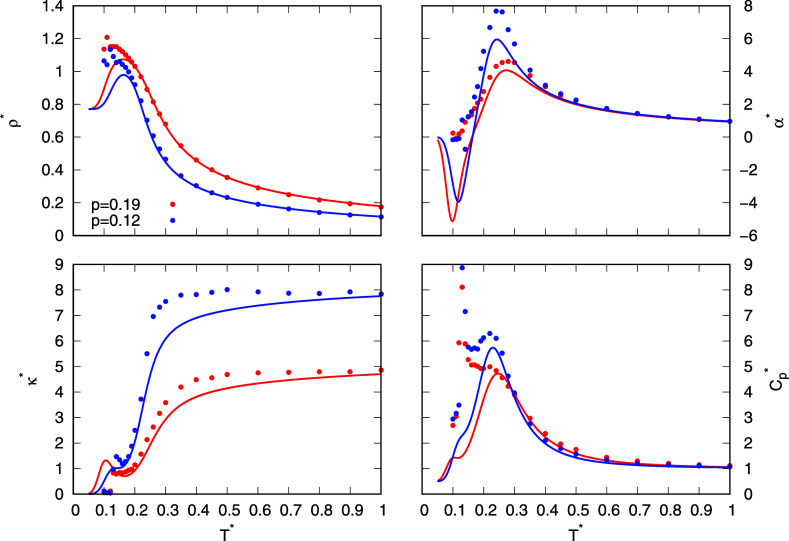
Thermodynamic properties (density, thermal expansion coefficient, isothermal compressibility, and heat capacity) as functions of temperature at pressures 0.19 and 0.12. MB-rose parametrization is used for analytical model and its results are plotted with lines, while results from MC simulations are plotted with points.

**Table 1:** Table of Parameters Used in Analytical Model for Both Parametrizations

	ε_HB_	ε_LJ_	*k*_s_	ε_c_	*r*_HB_	*x*_v_	σ_LJ_	*a*	*C*	*m*_1_	*m*_3_	*q*	*A*	*k*_HB_	*k*_LJ_	*k*_0_
MB-rose	1	0.1	16	–0.05	1	1.0	0.7	0.06	0.5	1.0	0.03675	3π/2	0.65	2*k*_s_	10	0.001
real-rose	1	0.2	6	0.06	1	1.15	0.891	0.06	0.4	1.0	0.03675	3π/2	0.475	2*k*_s_	16	0.001

## 3 Results and Discussion

All results are given in reduced units. The HB energy parameter ε_HB_ is used to normalize the temperature and the excess internal enthalpy ( $A^{*}=\frac{A}{\left|\varepsilon_{HB}\right|},T^{*}=\frac{k_{B}\times T}{\left|\varepsilon_{HB}\right|}$ ), distances are normalized by the characteristic length of the HB *r*_HB_ ( $r^{*}=\frac{r}{r_{HB}}$ ). The pressure in reduced units is defined as $p^{*}=\frac{pr_{HB}^{2}}{\left|\varepsilon_{HB}\right|}$ .

### 3.1 Thermodynamic Properties

Starting with thermodynamic properties of the analytical model, which is parametrized to mimic the two parametrizations of the rose water model, Figures 4 and S3 show comparisons of different thermodynamic properties as functions of temperature calculated with the analytical model and the MC simulations. The calculated and compared properties are density, thermal expansion, and heat capacity. As mentioned in the section describing the model, the parameters of the model were determined by fitting the density of the analytical model to the density obtained from the simulations. It is therefore expected that the agreement between the densities calculated with both methods will be good.

The thermodynamic properties of the rose model with MB parametrization are shown in Figure 4. As expected, the density predicted by the theory perfectly matches the density calculated by simulations, but only at temperatures above 0.20. The reason for this is that the density from the theory was fitted to the density from the simulations only in the temperature ranges where water is expected to be liquid or gas. The model was not fitted to the density of the solid, as the UD model was mainly developed for liquid water. In the UD model, the solid state is mainly represented by the *s* population, which assumes a perfect hexagonal lattice of water molecules, while in the simulations (and in reality), the structure of the solid phase can be more disordered, and some hexagonal interstitial sites are also filled with molecules. For this reason, the density of the analytical model at temperatures of around 0.1 is much lower than the density from simulations under the same conditions. Other thermodynamic properties of the model with MB-rose parametrization, which were calculated on the basis of the theory, are in semiquantitative agreement with the results of the simulations. The thermal expansion coefficient predicted by the theory reaches its maximum at the same temperatures as in the simulations but is slightly lower. Both methods also predict a minimum at a temperature slightly below the solid–liquid phase transition, although the theory’s minimum underestimates its depth. Overall, the agreement between the two approaches is very good, with almost perfect agreement at high temperatures. The isothermal compressibility of the theory agrees well with the simulations and shows the same qualitative trends but slightly lower values. At high temperatures, both methods give almost identical results. The first maximum in compressibility also corresponds to the solid–liquid phase transition. For the heat capacity, the agreement is very good, as both methods predict the same curve shape and almost agree quantitatively at temperatures above 0.2. The simulations show two maxima at temperatures around 0.12 and 0.2—both of which are reproduced by the theory. However, while the second peak agrees in height, the first is significantly underestimated, which is probably due to the limitations of the theory in representing the solid phase. The thermodynamic results of both the rose model and the analytical (rose-like) model are consistent with similar models—the MB model^48^ and the original UD model.^67^ Most of the large differences between the results obtained with the theory and the simulations in the low temperature range can be attributed to the poor representation of the solid phase by the theory. The solid phase as represented by theory is highly idealized, since at low temperatures most interactions are in an *s* state (Figure 5), which assumes an ideal hexagonal network of hydrogen bonds. However, in order to obtain more realistic results, the interstitial sites of the molecules should also be included. So coming back to the thermodynamic properties at low temperatures, the density predicted by the theory is too low, as the theory assumes a hexagonal lattice, while the solid in the simulations is less ordered and denser. The lower minimum of the predicted thermal expansion coefficient results from a larger volume change during the transition from the less dense solid phase to the liquid compared to the transition from the denser solid phase observed in simulations. Similarly, the first maximum of the heat capacity is lower in theory because the solid phase is too ordered in theory and, therefore, has fewer accessible degrees of freedom, resulting in a lower maximum at the phase transition. At these conditions, the rose model only has a solid phase with the structure of a hexagonal lattice. The melting of the solid phase is continuous, as the hexagonal structure is retained during the transition to the liquid phase, but it becomes more disordered.^69^ At a pressure of 0.19, the melting temperature of the rose water model with MB parametrization is 0.13 and with real parametrization 0.20. The theory successfully predicts the effects of pressure changes on the thermodynamic properties, as the thermodynamic properties calculated with the theory at a pressure of 0.12 also agree well with the properties calculated with simulations. The theory replicates the thermodynamic properties of the rose model with MB parametrization well at high temperatures and is generally consistent with it.

**Figure 5:**
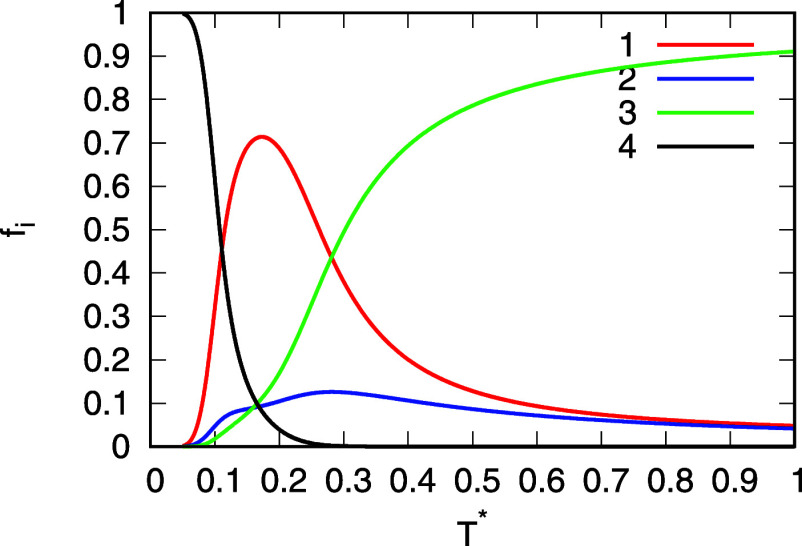
Populations of different interaction states as a function of temperature. MB-rose parametrization of the analytical UD model is used. Pressure in the system is 0.19. The populations are indexed by following numbers: 1-HB, 2-LJ, 3-0, and 4-s.

As already mentioned, the populations of the interaction states are important quantities that are calculated with the UD model and are decisive for the calculations of the structural properties presented later. Figure 5 shows these populations as functions of temperature for MB-rose parametrizations of the analytical model. The population of cooperative hydrogen bonds is represented by the number 4, normal/singular hydrogen bonds by the number 1, LJ contact by the number 2, and the noninteracting state by the number 3.

### 3.2 Dynamic Properties—Diffusion

Of the dynamic properties of water, the diffusion coefficient was calculated using an analytical model and compared with molecular dynamics (MD) simulations. Figure 6a,b shows the diffusion coefficient as a function of temperature at different pressures, while Figure 6c shows the diffusion coefficient as a function of pressure for different temperatures. The analytical model is very successful in describing the diffusion coefficient. The fit between the diffusion coefficient calculated with the theory and the simulations is practically perfect at the conditions where the parametrization of the analytical model was performed, i.e., at a pressure of 0.19 and temperatures around 0.2 (Figure 6a). The only region where a noticeable deviation between diffusion from the theory and the simulations occurs is the low pressure region, as can be seen in Figure 6c. For all temperatures shown, the agreement between theory and simulations is very good at pressures above 0.08, while the diffusion coefficients start to differ at lower pressures. At lower temperatures, the lowest pressure at which the diffusion values of the two methods still agree is also lower. In Figure 6c, for example, the deviation between the methods at a temperature of 0.4 appears at a pressure of about 0.08, while at a temperature of 0.2, the deviation appears at a pressure of about 0.02. The conditions under which the deviation between the methods occurs and also the value of the diffusion coefficient indicate that the system is in a gaseous state there. Under these conditions, the value of the diffusion coefficient calculated by the theory exceeds the value of the coefficient from the stimulations. To summarize, the analytical model predicts the diffusion coefficient very well under most conditions, while under more extreme conditions, the results of theory and simulations differ slightly.

**Figure 6:**
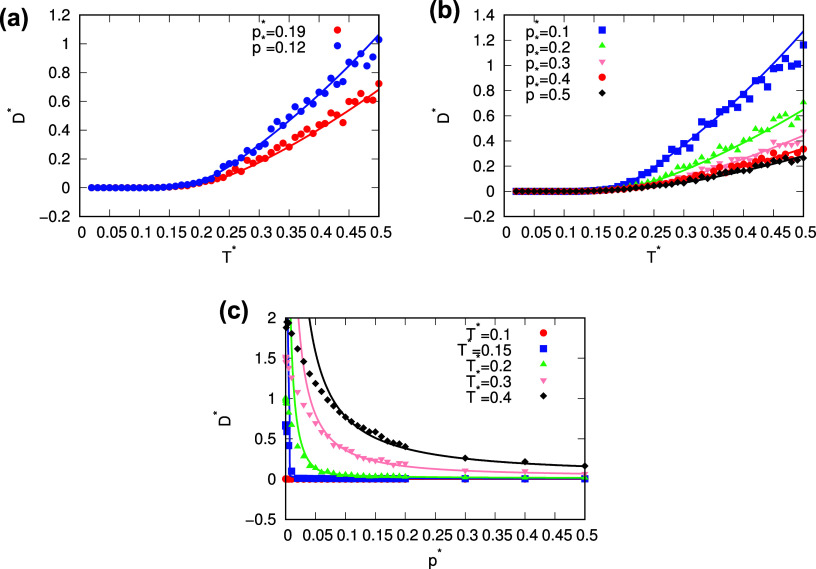
Diffusion as a function of temperature (a,b) and diffusion as a function of pressure (c). The MB-rose parametrization is used. Results from analytical model are plotted with lines and result from MD simulations are plotted with points.

### 3.3 Structural Properties

The focus of this study is the prediction of structural properties using a snowflake model developed by us.

The first structural property calculated with the snowflake model is the RDF between the water molecules. Figure 7 shows the RDF between the water molecules modeled with the rose model with MB parametrization at a pressure of 0.19 and different temperatures. Comparing the RDF calculated with the theory and the simulations, the overall agreement is very good. The theory successfully predicts both the positions and heights of all peaks, with some small differences. The agreement between the results of theory and simulations is very good at all temperatures shown. At temperature 0.15, however, the difference between the RDFs calculated with both methods is slightly larger than at the other temperatures. The difference is probably increased by the proximity to the solid state, and as already mentioned in the discussion of the thermodynamic results, the analytical model is not optimized for the solid state. Nevertheless, the positions and heights of the peaks in RDF at temperature 0.15 calculated with the theory agree well with the simulations. To better explain why some differences occur, we should first explain which structural patterns the main peaks in the RDF represent. The first peak at a distance of about 0.7 corresponds to two molecules in LJ contact; the second peak, the highest peak in Figure 7, corresponds to two molecules with direct HB between them. The peak at a distance of about 1.73 corresponds to the two molecules both hydrogen bonded to the third molecule. If you compare the RDF from theory and simulations in Figure 7, you will notice two differences in the peak heights. The first is the height of the first peak, which corresponds to the LJ contact. The height of this peak is too low according to the theory at low temperatures, while at higher temperatures, it is similar to that of the simulations, and at temperature 0.3, the height of the peak from the theory even exceeds that of the simulations. The reason why the first peak is too low at low temperatures is due to the initial assumption of the analytical model that the model has only three interacting sites and therefore can form LJ contacts with a maximum of 3 molecules, while the molecule in the simulations can form LJ contacts with more than 3 molecules simultaneously. Therefore, the probability of finding two molecules at a distance of 0.7 is higher in the simulations. On the other hand, at higher temperatures, the probability of finding a molecule at a distance of 0.7 becomes more and more similar in the simulations and in theory. This is because, in the snowflake model, molecules of higher shells can be placed close to the central molecule if the structure is disordered enough, which is the case at higher temperatures. Consequently, more than 3 molecules can be located within the distance of the LJ contact with the central molecule, while the central molecule initially has only three direct LJ contacts. As already mentioned, the main problem is that the theory does not take into account the interstitial sites of the molecules, which is directly reflected in the lowering of the first (LJ) peak at low temperatures. The second difference lies in the height of the second peak, which corresponds to a direct HB. The height of this peak predicted by theory is slightly lower than that predicted by the simulations. We believe that this is due to the possibility of hydrogen half-bond formation in the simulations, which increases the probability of finding two molecules at the HB distance in the simulations, since this distance is still favorable even if one of the molecules has an unfavorable orientation for HB. For further peaks (distance 1.73 and further), this effect of the half-bond disappears, as higher-order structures (not only direct interactions) cannot be formed by half-bonds alone.

**Figure 7:**
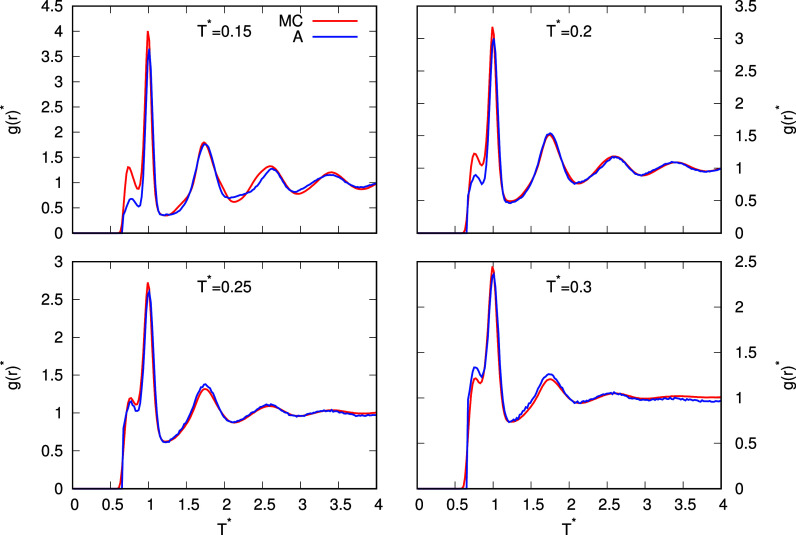
RDF between water molecules at different temperatures and pressure p* = 0.19. Results from MC simulations are plotted with red lines while results from the theory are plotted with blue lines. MB-rose parametrization of the model is used here.

The next structural property that was investigated is the angular distribution functions. The angular distribution functions were calculated for different radial distances, where each radial distance corresponds to a specific molecular arrangement in the structure. The distances at which the angular distributions were calculated are 0.7 (direct LJ contact), 1.0 (direct HB), 1.73 (three molecules connected with HB), and 2.0 (two molecules on the opposite side of the HB hexagon or the hexagonal interstitial sites of the second shell). A schematic representation of selected distances can be found in Figure 8. The angular distribution functions represent the position of the neighboring molecules in the body frame of the central molecule oriented such that the HB arm is at 0°. In other words, the angular distribution functions represent the angle between the HB arms of the central molecule and the line connecting the centers of the central and neighboring molecule. Figure 9 shows the angular distribution function for water modeled with the rose model with MB parametrization at different temperatures and a pressure of 0.19. The results of the analytical model and the simulations are compared. The overall agreement between the methods is quite good.

**Figure 8:**
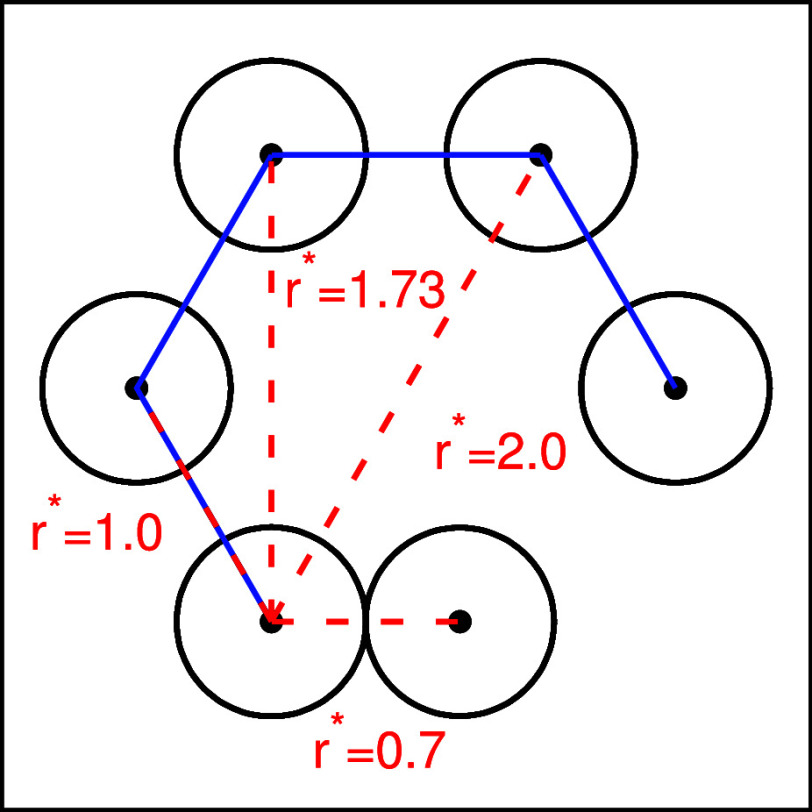
Schematic representation of the distances at which the angular distribution function is calculated. Blue lines represent HB between molecules and red dashed lines indicate selected distances.

**Figure 9:**
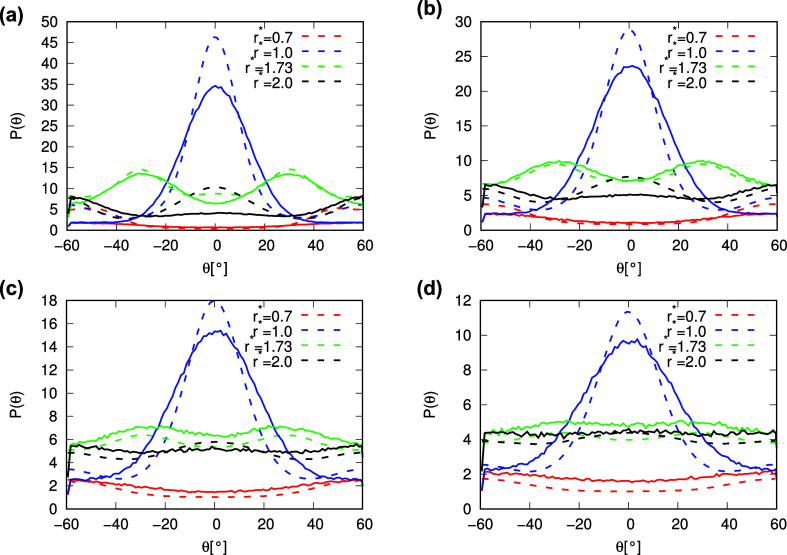
Angular distribution function between water molecules at pressure p* = 0.19 and different temperatures: (a) T* = 0.15, (b) T* = 0.2, (c) T* = 0.25, and (d) T* = 0.3. Results from MD simulations are plotted with dashed lines, while results from the theory are plotted with full lines. MB-rose parametrization of the model is used here.

Below you will find a brief description of the angular distributions and their relationship to the structure of the water. At a low temperature of 0.15 (Figure 9a), the structure of the water is very ordered, so that the peaks in the angular distributions are very pronounced. The angular distribution function at a radial distance of 0.7 represents the orientation of the molecules in the LJ contact. The simulations predict small maxima at angles −60° and 60° (which is actually one maximum due to 3-fold symmetry). This means that the probability of finding a molecule in the LJ contact is higher at the angles between the HB arms. In the ideal hexagonal structure, these positions are hexagonal interstitial sites. Thus, if all three arms are used for HB, three molecules can be placed between them and form an LJ contact with the central molecule. On the other hand, the analytical model does not predict this maximum because the molecule has only three interaction sites for all interactions. Thus, if the three interaction sites form three HBs, there can be no molecules between the arms that are in LJ contact with the central molecule. Furthermore, if all three interactions form LJ contacts, these LJ contacts are angle-independent; there are no angles that would be more favorable than the others. The next radial distance at which the angular distribution was calculated is 1.0, which corresponds to the HB length. As expected, the simulations predict a large maximum at angle 0°, which corresponds to the direct HB. In addition, the simulations also predict two small minima at the angles −60° and 60°. These two minima are the consequence of the formation of hydrogen half-bond formation, where the two molecules are at the distance of HB, while the neighboring molecule is oriented such that it can form HB and the central molecule is oriented such that it cannot form HB. The analytical model also successfully predicts the main maximum at angle 0°. The maximum agrees well with the maximum from the simulations. However, the width of the maximum predicted by the analytical model is larger than that predicted by the simulations, while the height of the maximum predicted by the analytical model is smaller. The analytical model does not predict the maxima at the angles −60° and 60° because the formation of a hydrogen half-bond is not taken into account in the analytical model. The angular distribution function calculated with the simulations at a radial distance of 1.73 has two maxima at angles of −30° and 30°. These two maxima correspond to the angles at which molecules are located in the second shell of the ideal hexagonal lattice: these are the molecules that are bound to the same molecule to which the central molecule is directly bound with HB. At this radial distance, the agreement between the angular distributions calculated with the analytical model and the simulations is practically perfect. The last radial distance for which the angular distribution function was calculated is distance 2.0. At this distance, the simulations predict three peaks in the angular distribution. Two peaks at angles −60° and 60° correspond to the molecules located in the third shell of molecules around the central molecule, or in the case of an ideal hexagonal lattice, these are the molecules located on the opposite side of the hexagons in which the central molecule participates. The third peak is located at an angle of 0°. This peak corresponds to the positions of the hexagonal interstitial sites, and as shown in Figure 9, the occupation of these interstitial sites is quite high in the simulations. The analytical model successfully predicts the positions of the two peaks at angles −60° and 60°, while the agreement between the distributions calculated by the theory and the simulations is almost quantitative at these angles. However, the analytical model predicts no peak at angle 0°, which was expected for the same reasons as the lack of peaks at angles −60° and 60° at a radial distance of 1.0. These reasons are the fact that half-bonds are not included in the analytical model and that the molecules of the analytical model have only three direct interaction sites. Thus, the main difference between the analytical model and the rose model is the limitation of the analytical model that there are no direct interactions that would favor the occupation of the hexagonal interstitial sites. On the other hand, the occupation of the hexagonal interstitial sites in the rose model used for the simulations is somewhat too favorable due to the ability to form half-bonds. At higher temperatures (Figure 9b–d), the structure becomes increasingly disordered. The main structural patterns remain the same as those at lower temperatures, so there are no new peaks that cannot be found at lower temperatures, while the same peaks become less prominent with increasing temperature. The agreement between the angular distributions calculated with the analytical model and the simulations becomes qualitatively better as the structure becomes more disordered and averaged, making the peaks in the angular distribution functions less prominent.

The agreement between the angular distribution functions calculated with the theory and the simulations could be improved by separating *k*_HB_ in eq 11 into radial and angular harmonic coefficients of HB and adjusting the coefficients so that the angular and radial distribution functions are in very good agreement with the simulation results. However, we did not do this because we wanted to create a simple structural model of water based on the UD model. When the model was constructed, we tried to keep the number of parameters as low as possible while still making the model physically meaningful.

One advantage of our new snowflake model is that we can also use it to calculate spatial distribution functions between water molecules. The spatial distribution function can also be calculated using simulations, but the time required for the calculations would be many orders of magnitude longer than the time required for our model. Figures 10 and S8 show spatial distribution functions of water molecules modeled with snowflake model using MB-rose and real-rose parametrizations. The distributions are shown at a pressure of 0.19 and different temperatures. Spatial distribution functions combine information already presented with radial distribution functions and angular distribution functions. In addition, they can give us a complete picture of the structure of the system, as they are like statistically averaged snapshots of the system. Figure 10a shows the spatial distribution function of the MB-rose parametrization at a temperature of 0.15. In the center of the figure is the central molecule, and as we move further away from the center, there are three regions of high probability that represent molecules that form a direct HB with the central molecule. Between these three regions, there are low probability regions that correspond to the hexagonal interstitial sites of the first shell. As you can see, the probability for these interstitial sites does not increase because the model does not allow for direct interaction between the central molecule and the molecules in the interstitial sites. Even further away from the center, there is another circle with a high probability that represents a second shell with molecules that are indirectly bound to the central molecule via the molecules in the first shell. If we look more closely, we can see the positions of the six molecules in this shell. The further we move away from the center, the less the areas of higher and lower probability differ, as the structural order is lost with increasing distance. At higher temperatures (Figure 10b–d), the shells with higher probability become less prominent. The radial and angular order decreases as the structure becomes more averaged with an increasing temperature. If we could distinguish the positions of 6 molecules in the second shell at a temperature of 0.15, the same shell at a temperature of 0.3 looks like a circular belt with a slightly higher probability.

**Figure 10:**
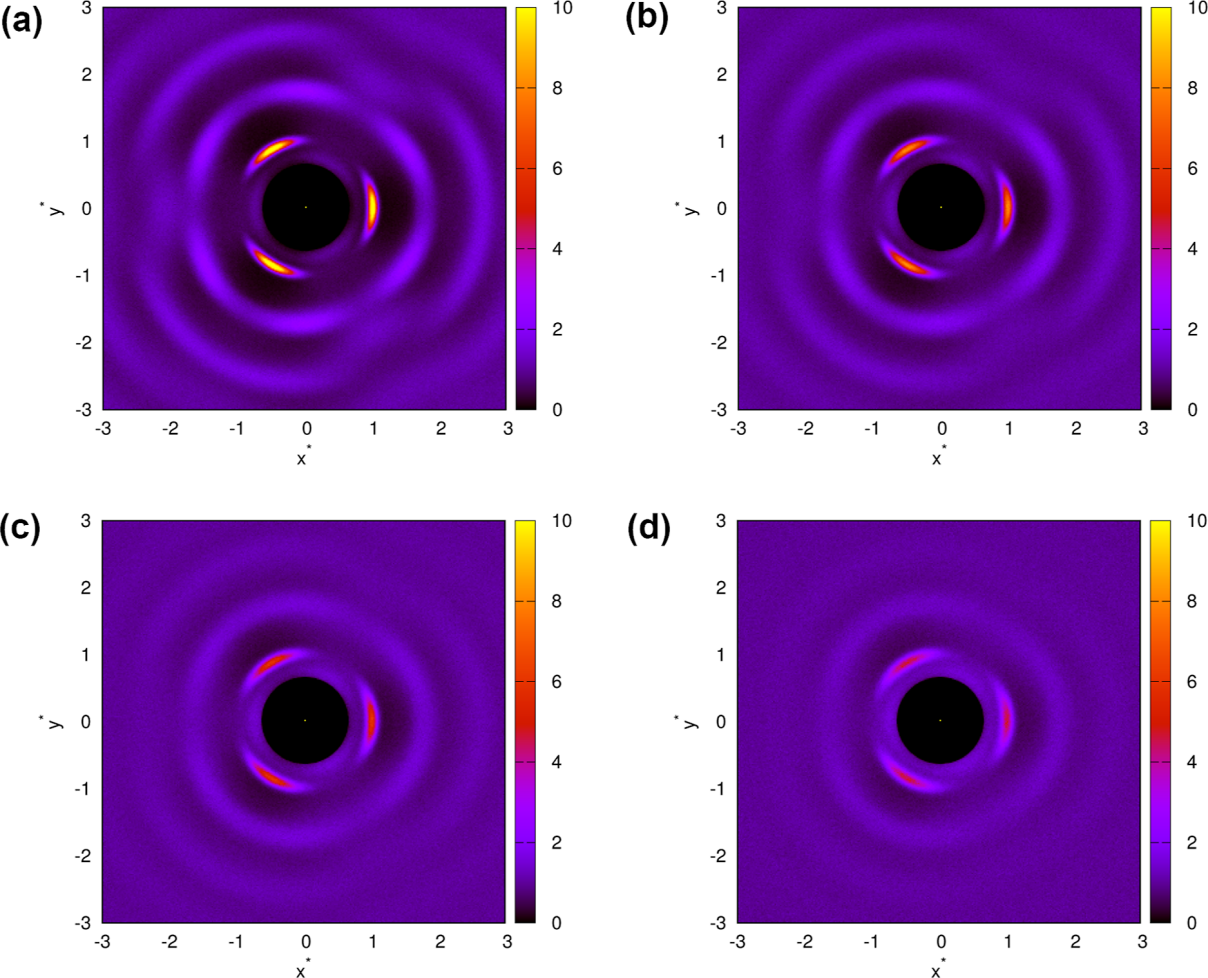
Spatial distribution function between water molecules at pressure p* = 0.19 and different temperatures: (a) T* = 0.15, (b) T* = 0.2, (c) T* = 0.25, and (d) T* = 0.3. The distribution is calculated using the theory with MB-rose parametrization of the model.

Overall, the analytical structural model allows us to calculate spatial distribution functions of water with an order of magnitude less computational time than simulations. These distribution functions can be used to directly observe the effects of different conditions and parametrizations on the structure of the water. The main difference between the analytical model and the rose model used in the simulation is that the analytical model does not allow direct occupation of interstitial sites. This leads to differences in the structure predicted by the theory and the simulation and is consequently reflected in differences in thermodynamic and dynamic behavior.

### 3.4 Computational Efficiency

To get a feeling for the computational efficiency of the analytical model we developed, the average computation times of MC, MD, and analytical model are compared in Table 2. For MC and MD, only the total simulation time is given, since the simulation time is relatively long and, in our programs, the sampling of the different properties was done during the simulation on the fly. For the analytical model, we divide the total time into different calculation sections, as these properties are calculated separately. Thermodynamic and dynamic properties are calculated with the UD model, and structural properties are calculated with the snowflake model using the results of the UD model. The table also shows the number of water molecules in the system used for the calculation of each method. For the structural part of the analytical model, the snowflake model, the number of molecules in the system depends on the conditions (temperature and pressure) in the system. For example, at low temperature, when the system is ordered, the density of the system is relatively low; therefore, the algorithm produces fewer molecules. At high temperature, on the other hand, there is less HB in the system, so the system is more disordered and denser, and the algorithm places more molecules in the system. As mentioned in the description of the snowflake model, the algorithm stops when the branches have no more room to grow or when the desired number of shells is reached. For the MC and MD simulations, we used in-house developed codes specifically designed for the rose water model (and the MB water model). The details of the simulations can be found in Section 2. The calculation times given in the table are the average time needed for the calculation in one phase point (*p*,*T*). The calculations were performed on a single core of an AMD EPYC 74F3 24-core processor. There is a large difference in the calculation time of MC and MD, which is due to the difference in sampling, as in MC, one particle is moved per step, while in MD, all particles are moved in each step. Comparing the calculation time of the analytical model with that of the simulations, the analytical model is still much faster than the simulations. The UD model part of the analytical model, which is used for the calculations of the thermodynamic and dynamic properties, is practically completely analytical, so that the calculations are completed almost instantaneously. The snowflake model, on the other hand, which is used to calculate the structural properties, takes more time to calculate because the model builds up the representations of the structures and is not truly analytical in the same sense as the UD model. Nevertheless, the new snowflake model is a fast approach for calculating the structural properties of water.

**Table 2:** Comparison of Computational Efficiency for MC, MD, and the Analytical Model^a^

	Monte Carlo (MC)	molecular dynamics (MD)	analytical model
total time	21 h 41 min	31 min	182 s
TD and *D* time			3.9 × 10^–5^ s
structure time			182 s
number of molecules	100	200	200–1600

a The total time represents the average total calculation time of the method, the TD and D time represent the time required to calculate the thermodynamic and dynamic properties, and the structural time represents the time required to calculate the structural properties. The number of molecules in the system used for the calculation is also included.

## 4 Conclusion

To conclude, the main objective of this work was to develop a structural model based on the ideas of the analytical UD water model and use it to calculate the structural properties of water. In the first part of this paper, the UD model was adapted so that its thermodynamic properties resembled the properties of the rose water model with two different parametrizations. The rose water model is a simple 2D water model in which the molecules are represented as LJ discs with an additional hydrogen bonding potential in the form of rose functions (combinations of sine functions). In the UD model, each molecule is modeled as a 2D disc with three equivalent interacting sites/arms, where each interacting arm can form three types of interactions, namely, HB, LJ contact, and no interaction at all. In addition, the distribution function of the model also includes a certain degree of hydrogen bonding cooperativity. The parametrization of the analytical UD model was carried out in such a way that its density was fitted to the density of the rose water model calculated by simulations. The thermodynamic properties calculated with the analytical model agreed well with the properties of the rose model calculated by simulations, with better agreement for the MB-rose parametrization than for real-rose parametrization. The analytical model was also used to calculate the diffusion coefficient of the water. In the analytical model, the diffusion coefficient is calculated based on populations of different types of interactions and characteristic distances between these interactions. The diffusion coefficient calculated with the analytical model was in excellent agreement with the diffusion coefficient obtained by simulations for both parametrizations of the model. Our new snowflake model was used to calculate the structural properties of water. The model uses the construction of snowflake-like structures that serve as an approximation of the molecular structure of water. The same basic assumptions are used here as for the UD model so that the models complement each other. The parametrization of the model is therefore mainly carried out in the thermodynamic part, while the structural model uses the same parameters and properties that were calculated in the thermodynamic part of the analytical model. Radial and angular distribution functions were calculated with the snowflake model and compared with the results of the simulations with the rose water model. The structural model was very successful in describing the radial distribution functions of the rose water model, as the agreement was practically quantitative. In addition, it was also successful in calculating the angular distribution functions, which agreed well with the results of the simulations, with some minor differences. The only major difference between the analytical model and the rose model used in the simulations is how favorable the occupation of the hexagonal interstitial sites is. Due to the ability of the rose model to form hydrogen half-bonds, the occupation of the hexagonal interstitial sites in the water structure is quite favorable. This is even more pronounced in the real parametrization of the rose model, in which the LJ potential minimum and the HB length are equal. On the other hand, the analytical model assumes that each molecule has only three interaction sites, so that the direct interaction with molecules in hexagonal interstitial sites is not taken into account, which makes the occupation of hexagonal interstitial sites less favorable. The snowflake model was also used to calculate the spatial distribution functions of the water molecules. These distribution functions give us direct insight into the structure of the system, in our case water, as they serve as a statistically averaged snapshot of the system.

All in all, we have successfully adapted the UD model to reproduce the thermodynamic and dynamic properties of the rose water model. In addition, the snowflake model was introduced, which allows the calculation of all structural properties of water in fractions of the computational time of the simulations. The principles and approach used here for the semianalytical modeling of liquid water could be applied to more complex systems in the future, starting with aqueous solutions of various solutes.

## Supplementary Material



## Notes

Rose model and analytical UD model; MC and MD simulations; effect of *k*_0_ on RDF; and real-rose parametrization of analytical model (PDF)
