# Supplementary material for: Snowflake Model of Water: A Fast Approach for Calculation of Structural Properties of Liquid Water
Source: J Chem Theory Comput. 2025 May 12;21(13):6337–51. doi: 10.1021/acs.jctc.5c00158 (PMC12243085; doi:10.1021/acs.jctc.5c00158)
Supplement: Supplementary file 1 [file ct5c00158_si_001.pdf]

Snowflake model of water: a fast approach for calculation of structural properties of liquid water.
SUPPORTING INFORMATION

Peter Ogrin and Tomaz Urbic*
Faculty of Chemistry and Chemical Technology,
University of Ljubljana, Vecna Pot 113, SI-1000 Ljubljana, Slovenia
(Dated: April 17, 2025)

S1. THE MODEL AND METHODS

A. Rose model

In this study, we used rose water model[1] as the reference water model used in the simulations. The rose water model is a simple two-dimensional model of water in which the molecules are represented by Lennard-Jones disks. In addition, the model includes a hydrogen bonding potential to account for the interactions between the molecules:

$$U(\vec{X_i}, \vec{X_j})_{rose} = U_{LJ}(r_{ij}) + U_{HB}(\vec{X_i}, \vec{X_j}), \tag{1}$$

where r_{ij} denotes the distance between the centers of the molecules i and j and $\vec{X_i}$ and $\vec{X_j}$ are vectors representing the positions and orientations of the molecules i and j respectively. The Lennard-Jones (LJ) potential has the standard form:

$$U_{LJ}(r_{ij}) = 4\epsilon_{LJ}\left[\left(\frac{\sigma_{LJ}}{r_{ij}}\right)^{12} - \left(\frac{\sigma_{LJ}}{r_{ij}}\right)^{6}\right]. \tag{2}$$

Both molecules in an interacting pair contribute to the total HB energy of the interaction, whereby the HB energy contribution of each molecule is independent of the other:

$$U_{HB}(\vec{X_i}, \vec{X_j}) = U_{HB}(\vec{r_{ij}}) + U_{HB}(\vec{r_{ji}}). \tag{3}$$

This independence allows the formation of "half" hydrogen bonds, where one molecule is ideally oriented with its arm towards the other molecule, while the other molecule is oriented differently. Although it is energetically less favorable than a full bond, a half bond can still form if the conditions are right. Conversely, if the second molecule cannot form a hydrogen bond, a half bond will always form because it is energetically more favorable than an LJ contact. The half-bond has no real physical meaning, they are an artefact of the potential.

The energy contribution of a molecule to the hydrogen bonding potential is the product of two terms: one depends on the orientation ($U(\theta_{ij})$) and the other on the distance ($s(r_{ij})$). The total energy contribution of a molecule is as follows :

$$U_{HB}(\vec{r_{ij}}) = \frac{\epsilon_{HB}}{2} \cdot s(r_{ij}) \cdot U(\theta_{ij}), \tag{4}$$

* tomaz.urbic@fkkt.uni-lj.si

where $\vec{r_{ij}}$ is the vector between the molecules i and j oriented in the body frame of the molecule i, where r_{ij} is the length of the vector and θ_{ij} is the angle of the vector in the body frame of the molecule i. The parameter ϵ_{HB} indicates the maximum energy of the hydrogen bond.

The orientational term of the HB potential depends on the relative orientation between the molecules. More precisely, from the perspective of molecule i, it depends on the position of molecule j in the body frame of molecule i. The orientation term is expressed by a combination of sine functions, called the rose function. For our model we use the 3-petal rose function:

$$U(\theta_{ij}) = a_2 \sin^2(3\theta_{ij}) + a_1 \sin(3\theta_{ij}), \tag{5}$$

where a_1 and a_2 are coefficients that control the shape of the potential as a function of the angle. This function, which defines the dependence on the orientation, gives the rose water model its name. To facilitate its use in simulations, the same function can be rewritten in Cartesian coordinates:

$$U_{HB}(\vec{r_{ij}}) = \frac{\epsilon_{HB}}{2} \cdot s(r_{ij}) \cdot \left(a_2 \cdot \frac{(3x_{ij}^2 y_{ij} - y_{ij}^3)^2}{r_{ij}^6} + a_1 \cdot \frac{3x_{ij}^2 y_{ij} - y_{ij}^3}{r_{ij}^3} \right), \tag{6}$$

where x_{ij} and y_{ij} are the Cartesian coordinates of the molecule j in the body frame of the molecule i, and $r_{ij} = \sqrt{x_{ij}^2 + y_{ij}^2}$.

Ideally, when the interacting molecules are in the position that maximizes their interaction (i.e. when the arms of the potential are parallel and aligned with the centers of the molecules), the value of the orientational term is -1. To normalize the interaction energy, the factor $\frac{1}{2}$ is inserted into the equation 4.

To model the distance dependence of the interaction, we use a double-sided cubic switching function:

$$s(r_{ij}) = \begin{cases} 0, & r_{ij} < r_l \\ \frac{(r_l+2r_{ij}-3r_{HB})(r_l-r_{ij})^2}{(r_l-r_{HB})^3}, & r_l \leq r_{ij} < r_{HB} \\ \frac{(r_u+2r_{ij}-3r_{HB})(r_u-r_{ij})^2}{(r_u-r_{HB})^3}, & r_{HB} \leq r_{ij} < r_u \\ 0, & r_u \leq r_{ij} \end{cases}$$

where r_{HB} is the hydrogen bond distance, and r_l and r_u represent the lower and upper limits for the hydrogen bond range. The switching function is symmetric, with $|r_{HB} - r_l| = |r_{HB} - r_u| = r_{FWHM}$, where r_{FWHM} is the "full width at half maximum" of the peak formed by the function.

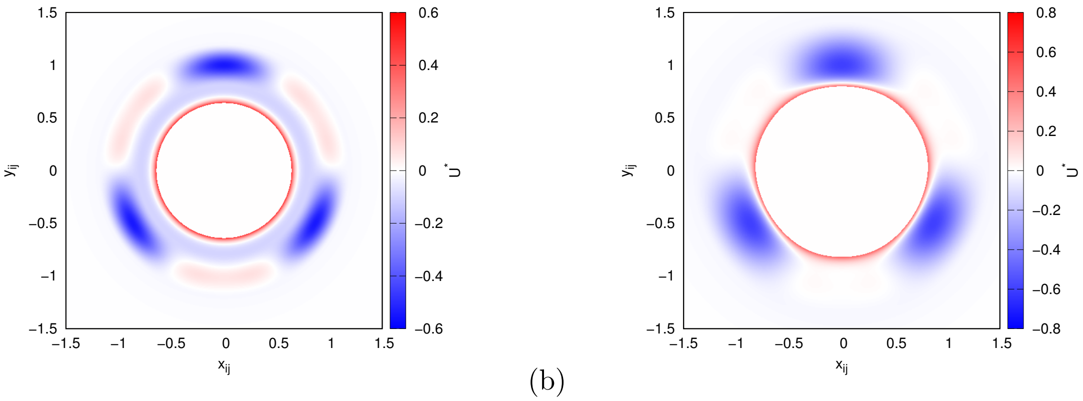

(a) (b)

Figure S1: Potential energy surface of rose water model with (a) MB parametrisation and (b) real parametrisation.

In this work we use two different parameterizations of the rose model. The model was originally developed as an imitation of the Mercedes-Benz (MB) water model[2, 3], and the first parameterization was chosen to approximate the properties of the MB model. This parameterization is referred to as the MB parameterization throughout the text. The model parameters are as follows: $\epsilon_{LJ} = 0.1$, $\sigma_{LJ} = 0.7$, $\epsilon_{HB} = 1$, $r_{HB} = 1$, $r_{FWHM} = 0.2$, $a_1 = 0.6$, and $a_2 = -0.4$. The Lennard-Jones potential in this parameterization is identical to that used in the MB model. We used the same parameters as in most previous studies on rose water model. The parameters in the MB parameterization of the rose model are chosen so that the (thermodynamic) properties of the rose model match the properties of the MB model as closely as possible. The second parameterization, which we refer to below as the real parameterization, was developed to correct certain exaggerations in the MB parameterization and to make the properties of the model more realistic. In particular, the distances between the interacting particles are adjusted so that the minimum of the Lennard-Jones potential coincides with the length of the hydrogen bond. In addition, the strength of the Lennard-Jones interaction is increased compared to the MB parameterization. The main differences in the real parameterization include: $\epsilon_{LJ} = 0.2$, $\sigma_{LJ} = 0.890899$ and a

larger hydrogen bond width, with $r_{FWHM} = 0.41666$. All other parameters remain the same as in the MB parameterization. The processes used to determine the parameters for these two parameterizations are described in detail in previous articles[1, 4].

B. Analytical model

1. *Statistical mechanics of the model*

The complete and detailed description of UD model can be found in[5]. The isothermal-isobaric statistical weight of the interaction states is calculated in a following way:

a. The hydrogen-bonded state To obtain the isothermal-isobaric partition function of the HB state, Δ_{HB}, the Boltzmann factor of the HB energy is integrated over all possible angles and distances between the central molecule and the neighbouring molecule. The integration results in the following partition function:

$$\begin{aligned}\Delta_{HB} =& c(T) v_{ef}^{HB} \exp\left(\frac{\epsilon_{HB} + \epsilon_{LJ} - 2pv_{HB}/3}{k_B T}\right) \\ &\times \sqrt{\frac{k_B T \pi}{k_s}} erf\left(\sqrt{\frac{k_s \pi^2}{9 k_B T}}\right),\end{aligned} \tag{7}$$

where $c(T)$ is the two-dimensional kinetic energy contribution, k_B is the Boltzmann constant, T is the temperature, p is the pressure, v_{HB} is the volume per molecule in the HB state and v_{ef}^{HB} is the effective volume resulting from the translational freedom between the two molecules. The volume per molecule in the HB state is determined by a simple geometric calculation. If water molecules form a perfect hexagonal crystal in which each molecule is connected to three neighbouring molecules by HB, this represents a low-pressure ice and its volume is equal:

$$v_s = \frac{3\sqrt{3} r_{HB}^2}{4} \tag{8}$$

, where r_{HB} is the HB length. The low-pressure ice has empty hexagonal vacancies - interstitial sites between the molecules. However, if these interstitial sites are also filled with water molecules, we obtain high-pressure ice with volume:

$$v_{HB}^{hp} = \frac{\sqrt{3} r_{HB}^2}{2}. \tag{9}$$

In the case of liquid water, the density of the water must lie between the densities of these two types of ice, so we use the following volume per molecule for liquid water:

$$v_{HB} = \frac{\sqrt{3} r_{HB}^2 x_v}{2}, \tag{10}$$

where x_v is a simple empirical factor that scales the density, for example by fitting it to the simulation data. The main purpose of the current model is to describe the liquid state. For this reason, we did not focus on different solid phases and only used the ideal hexagonal HB lattice as the ice phase.

b. The vdW/LJ contact state The isothermal-isobaric partition function of the LJ state, Δ_{LJ}, is calculated in a similar way as for the HB state by integrating the Boltzmann factor over the angles and positions. After integration, the partition function is

$$\Delta_{LJ} = \frac{2\pi}{3} c(T) v_{ef}^{LJ} \exp\left(\frac{\epsilon_{LJ} - 2pv_{LJ}/3}{k_B T}\right), \tag{11}$$

where v_{ef}^{LJ} is the effective volume of the LJ state resulting from translation, and v_{LJ} is the volume per molecule in the LJ state, which is

$$v_{LJ} = \frac{\sqrt{3}\sigma_{LJ}^2 \sqrt[3]{2}}{2}. \tag{12}$$

c. The noninteracting state After integrating the Boltzmann factor of the energy, the resulting isothermal-isobaric partition function is

$$\Delta_0 = \frac{2\pi}{3} c(T) \frac{k_B T}{p} \exp\left(\frac{-2pv_0/3}{k_B T}\right), \tag{13}$$

where v_0 is the volume per molecule, calculated as

$$v_0 = \frac{k_B T}{p} + v_{LJ}. \tag{14}$$

The mean field attraction energy beyond pair interactions is also assumed to be $-Na/v$, where a is the vdW dispersion parameter and v is the average molar volume[5–7].

2. Model for the dynamic properties

The average energies of interaction states are as follows

$$\langle u_{HB} \rangle = -\epsilon_{HB} - \epsilon_{LJ} + \frac{k_B T}{2} - \frac{\sqrt{k_s \pi k_B T} \exp\left(-\frac{k_s \pi^2}{9 k_B T}\right)}{3\mathrm{erf}\left(\sqrt{\frac{k_s \pi^2}{9 k_B T}}\right)} \tag{15}$$

$$\langle u_s \rangle = \langle u_{HB} \rangle + \frac{\epsilon_c}{6} \tag{16}$$

$$\langle u_{LJ} \rangle = -\epsilon_{LJ} \tag{17}$$

$$\langle u_0 \rangle = 0. \tag{18}$$

Once the diffusion coefficient has been determined, other dynamic properties such as viscosity, thermal conductivity and thermal diffusivity can also be calculated. A detailed description of how these are calculated can be found in a previous article[8].

3. Effect of k_0 on RDF

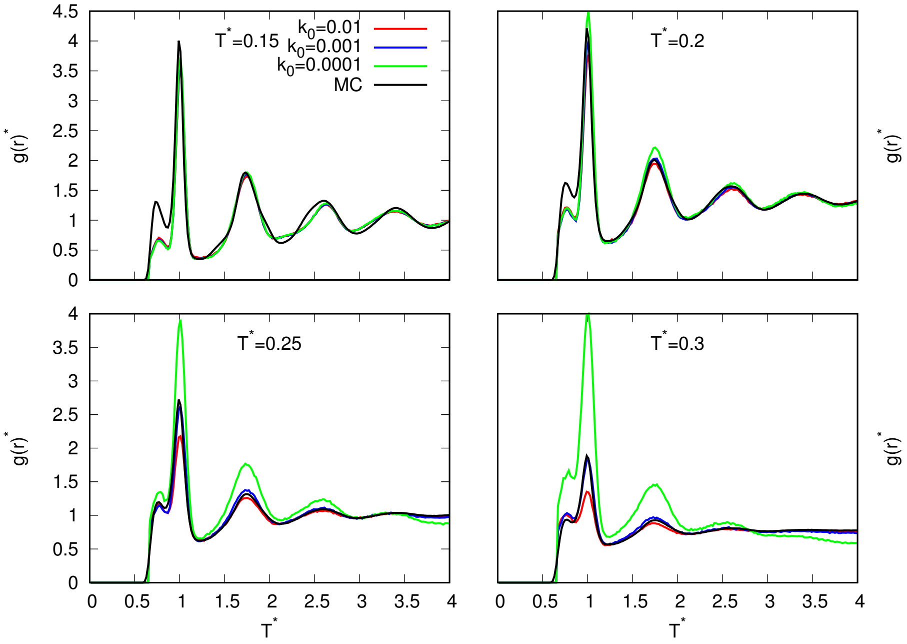

Figure S2: Effect of different coefficient k_0 on RDF. If the coefficient is to low the RDF decreases at larger distances when temperature is high.

Fig. S2 shows how RDF changes when the coefficient of the random motion potential of the non-interacting state (k_0) changes. If the coefficient is too low, the RDF decreases with distance at high temperature, while if it is too high, it disturbs the relationship between the HB and LJ states (for example, the peak at distance 1.0 becomes too low).

C. Monte Carlo simulations

The water model used in both the Monte Carlo and molecular dynamics simulations was rose water model[1, 4].

Monte Carlo simulations were performed using the Metropolis algorithm to determine the structural and thermodynamic properties of the rose water model using the two parameterizations described above. The results of these simulations were used as reference data for comparison with the results of the analytical theory. The simulations were performed in the NpT ensemble. Initially, 100 rose water particles were randomly placed in a square unit cell with a minimum distance between particles of at least σ_{LJ} to avoid particle overlap. In order to simulate macroscopic systems, periodic boundary conditions with the minimum image convention were applied.

In each simulation step, a random rotation and a random translation of a randomly chosen molecule were attempted. Consequently, each simulation cycle included N translation and N rotation trials. To keep the pressure constant, an attempt was made to adjust the volume of the system once per cycle. The equilibration phase consisted of 100,000 cycles, followed by a sampling phase in which ten series of 100,000 cycles each were performed to calculate the structural and thermodynamic properties. In addition, some simulations were performed with 200 particles to ensure that the results were not influenced by system size effects.

D. Molecular dynamics simulations

Molecular dynamics (MD) simulations were used to calculate the properties of the rose water model, which were then used as a reference for comparison with the results of the analytical model. The MD simulations were performed using an in-house MD code[9], originally developed for the MB water model. However, given the similarity between the MB and Rose models, the interaction potential in the code was changed to the rose model. The simulations were performed in the NpT ensemble, applying periodic boundary conditions and the minimum image convention to replicate a macroscopic system.

The velocity Verlet algorithm[10], with a time step of 0.001 ($t^* = t\sqrt{\frac{\epsilon_{HB}}{mr_{HB}^2}}$) was used to integrate the trajectories. The system underwent an equilibration of 100,000 steps followed by a sampling phase during which various structural, thermodynamic and dynamic variables

were samplet. The sampling phase consisted of 20 separate series, each comprising 100,000 steps. Throughout the simulation, the system contained 200 water molecules, with the initial configuration randomly generated while also ensuring that the molecules did not overlap.

During the equilibration phase a simple velocity rescaling thermostat was used to scale the velocity, while in the sampling phase a stochastic velocity rescaling method[11] was used to scale the velocity. The Berendsen barostat[12] was applied during equilibration, and a stochastic cell rescaling approach[13] was used during the sampling phase. The thermostat coupling constant was set to 0.01 and the barostat coupling constant was set to 0.1.

Thermodynamic, structural and dynamic properties were calculated using standard formulas. The angular distributions were determined by counting the number of molecules at different positions and plotting the resulting histogram.

S2. RESULTS AND DISCUSSION OF REAL PARAMETRIZATION

A. Thermodynamic properties

Problem of rose model with real parametrization Fig. S3 shows the comparison of the thermodynamic properties of rose model with the real parameterization, which was calculated using the theory (analytical model) and MC simulations. The figure is identical to Fig. 4, except that a different parameterization of rose model is used. It can be seen immediately that the agreement between the simulations and the theory with the real parameterization is not as good as with the MB parameterization of the model, but the agreement here is still good. The analytical model for real parametrization was parameterized in the same way as for the MB parameterization. This means that the density of the liquid and gaseous state was calculated as a function of temperature using the theory and fitted to the density calculated using the simulations. In this parameterization, the melting point is at a higher temperature than in the MB-rose parameterization, where it is around temperature 0.2. Here, the difference between the solid state predicted by the theory and the solid state that occurs in the simulations is even greater and more pronounced. Here, too, the density of the solid state calculated by the theory is lower than in the simulations, as the theory assumes a perfect hexagonal lattice with empty vacancies, whereas in the simulations many of these vacancies are filled with molecules. If the real parameterization of rose model is

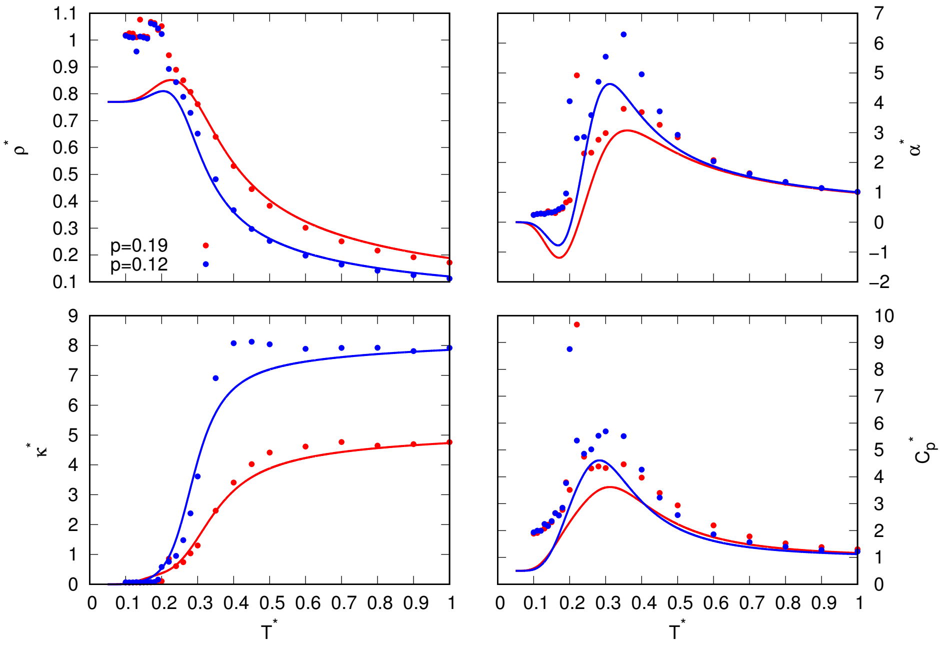

Figure S3: Thermodynamic properties (density, thermal expansion coefficient, isothermal compressibility and heat capacity) as functions of temperature at pressures 0.19 and 0.12. Real-rose parametrisation is used for analytical model and its results are plotted with lines, while results from MC simulations are plotted with points.

used, the occupation of the hexagonal vacancies by water molecules is even more favourable than when MB parametrisation is used. This is because the minimum of the LJ interaction is at the same distance as the length of the HB bond, which means that the most energetically favourable distance between the two molecules is always the distance of the HB length, regardless of the orientation of the molecules. Combining this with the ability of the rose model to form "half bonds", the placement of the water molecule in the hexagonal vacancy becomes quite favourable, as this molecule interacts with 6 neighbours with LJ contact and forms 3 hydrogen "half bonds" with 3 neighbouring molecules. Thus, to improve the analytical model and to better reproduce the properties of rose model with real parametrisation, another population of a high density solid should be included in the model, where the molecules are bonded with HB and the vacancies of the hexagonal lattice are filled. Why we did not do this? Because we wanted to keep the analytical model as simple as possible, and also because it would make the model less similar to the real physical water. In the model description, it was mentioned that the real parameterization

of rose model was developed because the MB parameterization had some properties that were exaggerated compared to the experimental water. A significant difference between the rose model with MB parameterization and the experimental water is that it has two "first" peaks (one corresponding to LJ contact and one corresponding to direct HB) in the radial distribution function (Fig. 7), while the experimental water has only one "first" peak[14]. This is corrected in the real parameterization of rose model by aligning the LJ potential minimum with the HB length. The two peaks in the MB-Rose parameterization are due to the two characteristic lengths of the LJ and HB interaction. They are not a consequence of the dimensionality of the model, as there are also two characteristic peaks in both the 2D and 3D versions of the MB model[15]. However, this alignment of the distances in combination with the formation of half-bonds leads to other unrealistic properties, such as a too favourable occupation of the hexagonal vacancies in the structure. For this reason, we have not changed the solid state of the analytical model so that it corresponds more to rose model with real parameterization. Now that the main problem with the real parameterization has been discussed, let us proceed with the discussion of the agreement of the thermodynamic properties of rose model with the real parameterization predicted by both methods. In Fig. S3 other thermodynamic properties such as the coefficient of thermal expansion, the isothermal compressibility and the heat capacity are compared. The agreement between the quantities calculated with the theory and the simulation is semi-quantitative here and becomes almost perfect in the high temperature range. The theory also correctly predicts the positions of all important extrema. However, similar to the MB parameterization, the maximum of the heat capacity, which corresponds to the melting of the solid, is too low - here it is practically undetectable when the real parameterization is used. The effect of the pressure change on the thermodynamic properties is also correctly reproduced by the theory. Overall, the agreement between the thermodynamic properties calculated with the theory and the simulations is very good for the real parameterization of the model, but somewhat less good than for the MB parameterization. A possible explanation for the slightly worse results is that the formation of half-bonds becomes more favourable with real parameterization, so that the population of half-bonds in the system, which the analytical model does not take into account, becomes large enough to have a noticeable influence on the properties.

Fig. S4 shows these populations as functions of temperature for both the real-rose parameterizations of the analytical model. Qualitatively, the populations show the same trends in

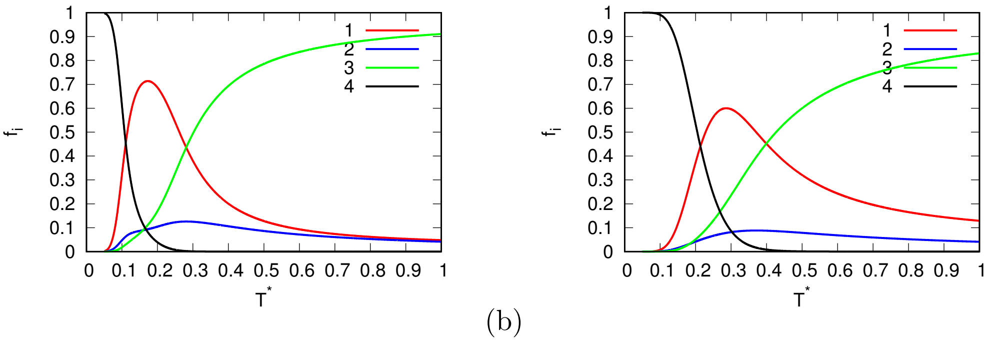

(a) (b)

Figure S4: Populations of different interaction states as function of temperature. In the left figure MB-rose parametrisation of analytical model is used while in the right one real-rose parametrisation is used. Pressure in the system is 0.19. The populations are indexed by following numbers: 1 - HB, 2 - LJ, 3 - 0, 4 - s.

the case of both parameterizations, but there are quantitative differences in the populations when a different parameterization is used. The main difference is that when using the Real-rose parameterization, all curves are shifted towards higher temperatures than when using the MB-rose parameterization. In practice, this means that the temperatures of the phase transitions are higher. Another difference is that when using the real parameterization, the population of LJ contacts is lower over the entire temperature range. This is logical as there are fewer pure LJ interactions (where there are no full or half hydrogen bonds) in the system.

B. Dynamic properties - diffusion

Of the dynamic properties of water, the diffusion coefficient was calculated using an analytical model and compared with MD simulations. Fig. S5 (a) and (b) show the diffusion coefficient as a function of temperature at different pressures, while Fig. S5 (c) show the diffusion coefficient as a function of pressure for different temperatures. The analytical model is very successful in predicting the diffusion coefficient for real parameterizations of the model.

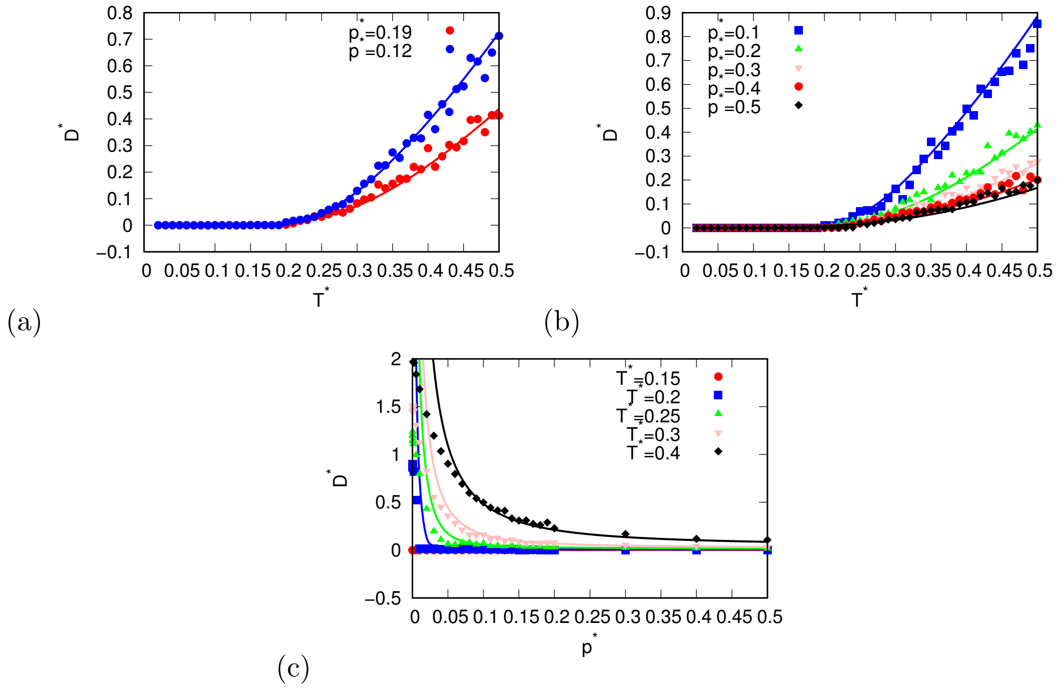

Figure S5: Diffusion as function of temperature ((a) and (b)) and diffusion as function of pressure (c). The real-rose parametrisation of the model is used. Results from analytical model are plotted with lines and result from MD simulations are plotted with points.

C. Structural properties

The radial distribution functions for the fit were calculated at a pressure of 0.19 and a temperature of 0.25 for the real parameterization.

In Fig. S6 radial distribution functions between water molecules modelled with the rose model with real parametrisation are shown. There is a significant difference between the RDF of the rose model with MB and the real parameterization, namely that in the real parameterization the two peaks that represented the LJ contact and the direct HB in the MB parameterization are now merged into one peak at the distance of the HB. For this reason, it is easier to evaluate the analytical model with the MB-rose parameterization because the characteristic distances of the two interactions are separated, whereas with the real-rose parameterization it is difficult to decide which interaction contributes to the change in the RDF. In Fig. S6, the temperatures at which RDFs are calculated are slightly higher than in Fig. 7 because the melting point of the model with real parameterization is slightly higher than that of the model with MB parameterization and we wanted to show

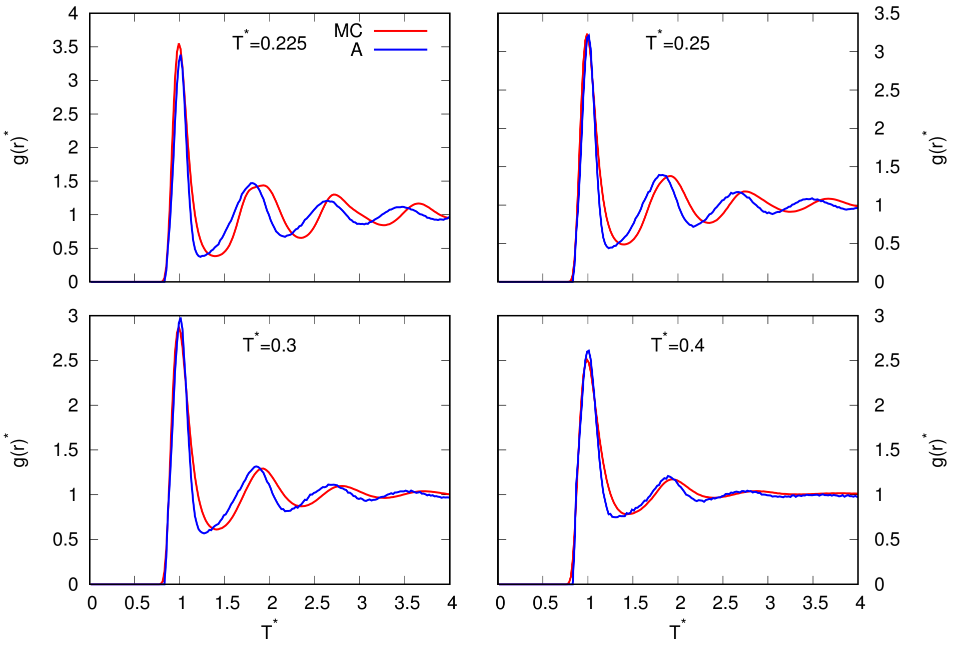

Figure S6: Radial distribution function between water molecules at different temperatures and pressure p^* =0.19. Results from MC simulations are plotted with red lines while results from the theory are plotted with blue lines. Real-rose parametrisation of the model is used here.

a similar temperature range in terms of properties. When comparing the RDF of the real parameterization calculated according to the theory and the simulations, it is immediately noticeable that the RDF calculated according to the theory appears to be shifted towards lower distances. Apart from this "shift", both the position and the height of all peaks in the RDFs match well between the theory and the simulations. The reason for the "shift" of some peaks is again due to the assumption of the analytical model that there are only three interaction sites, while on the other hand the real parameterization in the simulations even favours the formation of half-bonds, which often leads to more than three interacting molecules around each molecule. The alignment of the LJ and HB distances in the real parameterization favours the formation of half-bonds, which also lead to a more favourable occupation of the hexagonal vacancies in the water molecule lattice. This occupation of the hexagonal vacancies, or in other words the formation of a hexagonal dense package, is a direct reason for the difference in the position of the peaks calculated by the theory and the simulations in Fig. S6. If we have an ideal hexagonal lattice with a molecule size of 1.0, the

distance to the neighbours in the second shell of the central molecule (the molecule that is bound to the molecule that is bound to the central molecule) is 1.73 and to the third shell of the neighbours is 2.0. There are 6 molecules in the second shell and 3 molecules in the third shell, but there are also 3 hexagonal vacancies at a distance of 2.0. Since in Fig. S6 we do not have an ideal structure, the peaks at 1.73 and 2.0 in the analytical model are merged into a broader peak at a distance of about 1.8. In the case of the simulation, however, the positioning of the molecules in the hexagonal vacancies is more favourable and therefore the peak shifts towards larger distances. The same happens with further peaks. This is the explanation for the difference in the position of the peaks in Fig. S6. As the temperature increases, the difference between the peak positions from theory and simulation decreases because the structure becomes more disordered and the snowflake model indirectly fills the hexagonal vacancies of the hexagonal lattice, which is highly disordered due to the high temperature. Consequently, the theory predicts the same positions of the second and further peaks at high temperatures (e.g. temperature 0.4). In summary, the theory is very successful in predicting the RDF of the rose water model with real parameterization, because if we consider the initial assumption of the analytical model, the theory gives excellent results. However, due to the unrealistically favourable placement of the water molecules with real parameterization in the hexagonal vacancies in the simulations, the simulations yield slightly different positions of the RDF peaks.

Next, we consider the angular distribution functions of rose model with real parameterization shown in Fig. S7. At a low temperature of 0.225 (Fig. S7 (a)) (the lowest temperature in Fig. S7 is higher than in Fig. 9, because the model with real parameterization has a higher melting temperature), similar angular distributions are shown as in the MB parameterization. The main difference is that here we do not have an angular distribution at radial distance 0.7 because this distance cannot be achieved due to the larger excluded volume of the molecules - larger LJ distance parameter. At other radial distances, the angular distribution functions of the model with real parameterization have the same maxima as in the MB parameterization, which is to be expected since the formation of HB with threefold symmetry of the molecules is the basis for the ordering of the structure. In the angular distribution functions calculated with the simulations, the peaks corresponding to the molecules in the hexagonal vacancies are even more pronounced than in the MB parameterization, since in the real parameterization of the model the occupation of the hexagonal

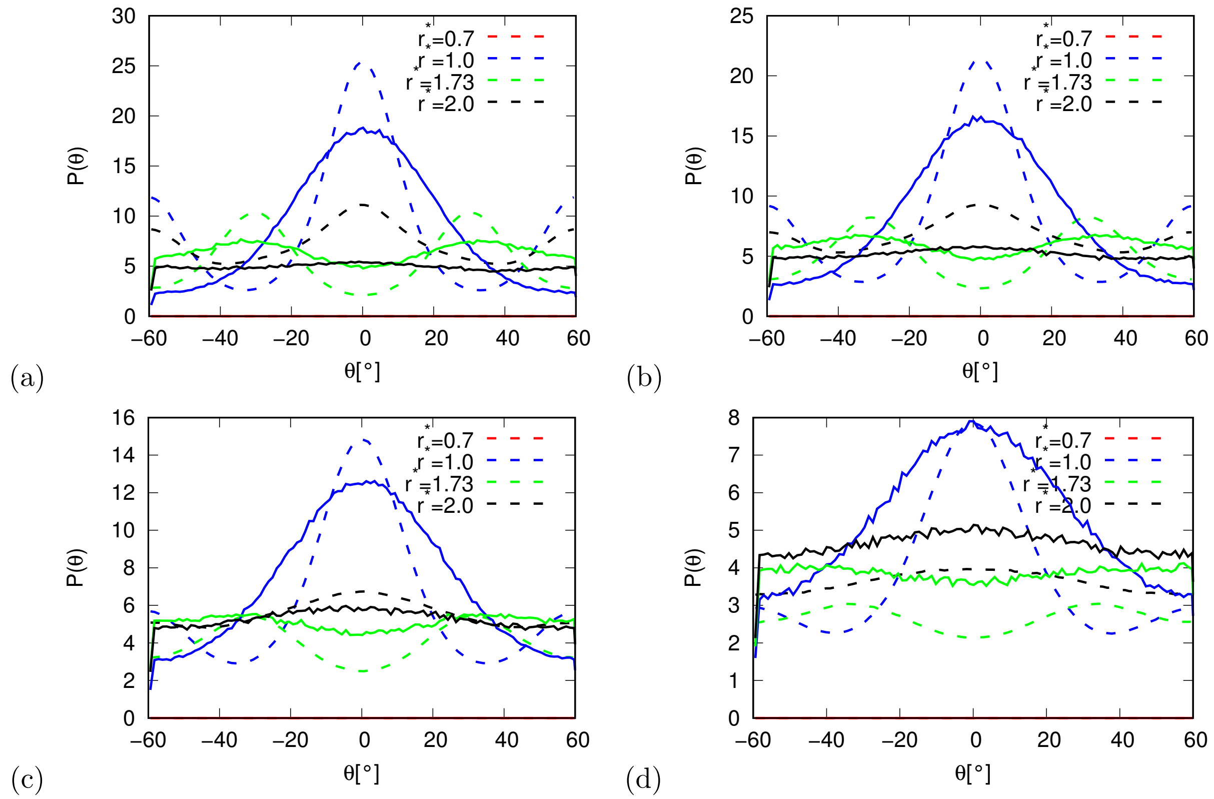

Figure S7: Angular distribution function between water molecules at pressure p^* =0.19 and different temperatures: (a) T^* =0.225, (b) T^* =0.25,(c) T^* =0.3,(d) T^* =0.4. Results from MD simulations are plotted with dashed lines while results from the theory are plotted with full lines. Real-rose parametrisation of the model is used here.

vacancies is even more favourable. On the other hand, the analytical model does not include direct interactions with the molecules in the hexagonal vacancies. Therefore, the agreement between the angular distribution calculated with the analytical model and the simulations becomes worse when the real parameterization is used instead of the MB parameterization. At higher temperatures (Fig. S7 (b), (c), (d)) the structure becomes more disordered and the peaks become less distinct. Another difference in the angular distribution calculated by the analytical model and the simulations shown in Fig. S7 is the width of the main peaks, as the peaks calculated with the analytical model (especially at a radial distance of 1.0) are wider than those calculated with the simulations. In the structural analytical model, the harmonic potential coefficient k_{HB}, which models the deviation of hydrogen bond from the ideal position (Eq. 11), is taken directly from the thermodynamic analytical model, where

k_s was determined by density fitting. The analytical model and the rose model used for the simulations have some differences in their basic structure (especially the limitation to three direct interaction sites in the analytical model and the ability to form half-bonds in the rose model) and have slightly different properties due to these differences. By fitting the analytical model to the density of the rose model calculated by simulations, some differences in the thermodynamics could be compensated, while at the same time the choice of parameters also influenced the structural properties of the analytical model. In Fig. S7, for example, we can see that the width of the main peak at a radial distance of 1.0 is larger when calculated with the analytical model. Perhaps this larger width is a compensation for empty hexagonal voids, and as such makes the thermodynamic properties of the analytical model more similar to the properties of the rose model.

Fig. S8 shows the spatial distributions of the model with real-rose parameterization. The effects of distance and temperature on the structure of the system are the same as for the MB-rose parameterization. Since the harmonic potential constant for HB, k_{HB}, is lower in the real-rose parameterization than in the MB-rose parameterization, the structure of the spatial distribution function is more angularly and radially averaged. At a temperature of 0.225 (which is slightly above the melting point - similar to the temperature 0.15 in the MB-rose parameterization), the positions of the molecules of the second shell can still be distinguished, but not as easily as in the case of the MB-rose parameterization at a temperature of 0.15. A significant difference between the spatial distribution functions for the real-rose and MB-rose parametrizations is that in the MB-rose parametrization there is a narrow belt with a slightly higher probability at the positions of direct LJ contact, while this belt does not exist in the real-rose parametrization. In the MB-rose parameterization, the probability of finding molecules in this belt increases with temperature, which means that the direct LJ contact becomes more favourable with increasing temperature. The same can be observed for the real-rose parameterization, where the probability decreases at a radial distance of 1.0 in the angles of the HB arms, while at the same time the probability increases in the angles between the HB arms.

---

[1] Williamson, C. H.; Hall, J. R.; Fennell, C. J. Two-dimensional molecular simulations using rose potentials. J. Mol. Liq. 2017. 228, 11–18.

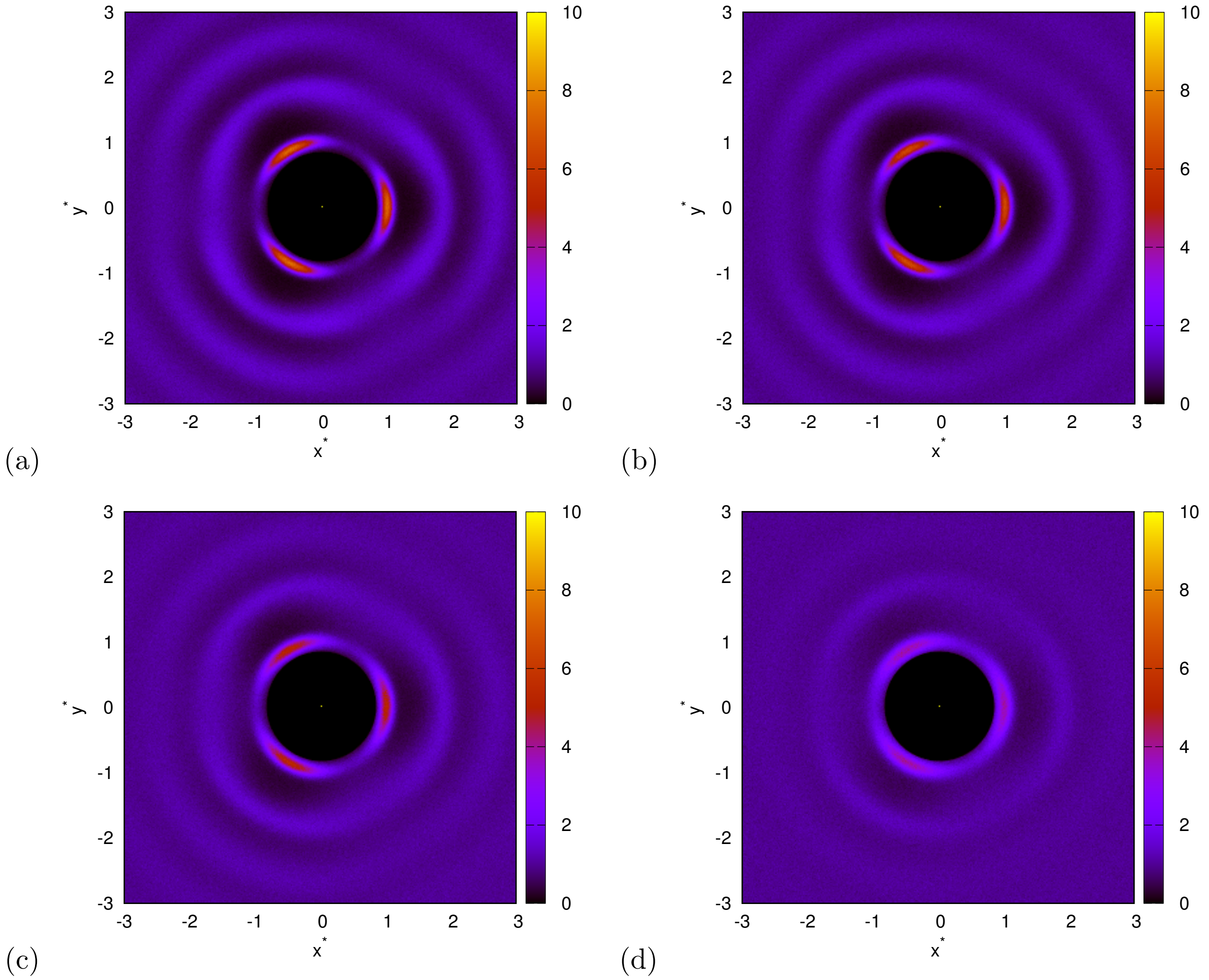

Figure S8: Spatial distribution function between water molecules at pressure p^* =0.19 and different temperatures: (a) T^* =0.225, (b) T^* =0.25,(c) T^* =0.3,(d) T^* =0.4. The distribution is calculated using the theory with real-rose parametrisation of the model.

[2] Ben-Naim, A. Statistical mechanics of ”waterlike ” particles in two dimensions. I. Physical model and application of the Percus-Yevick equation. The J. Chem. Phys. 1971. 54, 3682–3695.

[3] Silverstein, K. A.; Haymet, A. D.; Dill, K. A. A simple model of water and the hydrophobic effect. J. Am. Chem. Soc. 1998. 120, 3166–3175.

[4] Ogrin, P.; Urbic, T.; Fennell, C. J. Statistical-mechanical liquid theories reproduce anomalous thermodynamic properties of explicit two-dimensional water models. Phys. Rev. E 2022. 106, 034115.

[5] Urbic, T.; Dill, K. A. A statistical mechanical theory for a two-dimensional model of water.

The J. Chem. Phys. 2010. 132, 224507.

[6] Jagla, E. A. Core-softened potentials and the anomalous properties of water. The J. Chem. Phys. 1999. 111, 8980–8986.

[7] Truskett, T. M.; Dill, K. A. A Simple Statistical Mechanical Model of Water. The J. Phys. Chem. B 2002. 106, 11829–11842.

[8] Urbic, T.; Dill, K. A. Simple Model of Liquid Water Dynamics. The J. Phys. Chem. B 2023. 127, 7996–8001.

[9] Ogrin, P.; Dias, C. L.; Urbic, T. Code for molecular dynamics simulation of two dimensional Mercedes-Benz water model. Comput. Phys. Commun. 2024. 303, 109267.

[10] Swope, W. C.; Andersen, H. C.; Berens, P. H.; Wilson, K. R. A computer simulation method for the calculation of equilibrium constants for the formation of physical clusters of molecules: Application to small water clusters. The J. Chem. Phys. 1982. 76, 637–649.

[11] Bussi, G.; Donadio, D.; Parrinello, M. Canonical sampling through velocity rescaling. The J. Chem. Phys. 2007. 126, 014101.

[12] Berendsen, H. J. C.; Postma, J. P. M.; van Gunsteren, W. F.; DiNola, A.; Haak, J. R. Molecular dynamics with coupling to an external bath. The J. Chem. Phys. 1984. 81, 3684–3690.

[13] Bernetti, M.; Bussi, G. Pressure control using stochastic cell rescaling. The J. Chem. Phys. 2020. 153.

[14] Brookes, D. H.; Head-Gordon, T. Family of Oxygen–Oxygen Radial Distribution Functions for Water. The J. Phys. Chem. Lett. 2015. 6, 2938–2943.

[15] Bizjak, A.; Urbic, T.; Vlachy, V.; Dill, K. A. Theory for the three-dimensional Mercedes-Benz model of water. The J. Chem. Phys. 2009. 131.
